# Chinese Herbal Medicine Combined With Antiepileptic Drugs for Intractable Epilepsy: A Systematic Review and Meta-Analysis of Randomized Controlled Trials

**DOI:** 10.3389/fphar.2022.917099

**Published:** 2022-07-20

**Authors:** Ying Zhao, Hufang Zhou, Qingxuan Liu, Jialin Liu, Mengwei Wu, Siyuan Yuan, Weiwei Xu, Ying Wang, Kaiyue Wang, Lili Li, Jinmin Liu

**Affiliations:** ^1^ Dongfang Hospital, Beijing University of Chinese Medicine, Beijing, China; ^2^ Dongzhimen Hospital, Beijing University of Chinese Medicine, Beijing, China; ^3^ Beijing University of Chinese Medicine, Beijing, China

**Keywords:** intractable epilepsy, Chinese herbal medicine, antiepileptic drugs, systematic review, meta-analysis

## Abstract

**Background:** Intractable epilepsy (IE) is still a major concern in neurology, and existing therapies do not adequately control symptoms. Chinese Herbal Medicine (CHM) has been widely used as an adjunct to antiepileptic drugs (AEDs) for IE. However, because of the contradictory findings reported in previous studies, it is uncertain if the present evidence is robust enough to warrant its usage. The purpose of this meta-analysis was to systematically evaluate the efficacy of the combination of CHM and AEDs for IE.

**Methods:** From inception to September 2021, Medline, Ovid, Embase, Cochrane Library, Chinese Biomedical Database, China National Knowledge Infrastructure, VIP Database, and Wanfang Database were searched. Only randomized controlled trials (RCTs) that assessed the efficacy of the combination of CHM and AEDs for IE were included. We defined monthly seizure frequency as the primary outcome. The secondary outcomes included the abnormal rate of electroencephalogram (EEG), seizure duration, quality of life (QoL), and adverse events (AEs).

**Results:** Twenty studies with 1,830 patients were enrolled. Most trials had poor methodological quality. The meta-analysis showed that the combination of CHM and AEDs was more efficient than AEDs alone in reducing monthly seizure frequency [MD = −1.26%, 95% CI (−1.62, −0.91); *p* < 0.00001], the abnormal rate of EEG [RR = 0.66%, 95% CI (0.53, 0.82); *p* = 0.0002], and improving the QoL [MD = 6.96%, 95% CI (3.44, 10.49); *p* = 0.0001]. There was no significant difference in seizure duration between groups. Moreover, the combination of CHM and AEDs significantly reduced the AEs [RR = 0.45%, 95% CI (0.32, 0.64); *p* < 0.00001].

**Conclusion:** The combination of CHM and AEDs could improve seizure control by reducing monthly seizure frequency and abnormal rate of EEG with a decreased risk of adverse events in patients with IE. However, these findings must be interpreted carefully due to the high or uncertain risk of bias in the included trials. To provide stronger evidence for the use of CHM combined with AEDs in IE, high-quality RCTs will be urgently warranted in the future.

## Introduction

Epilepsy is a neurological disorder marked by recurrent seizures that affects over 70 million individuals worldwide (Ding et al., 2021). Epilepsy sufferers’ morbidity and mortality are affected by ongoing seizures. Patients with epilepsy are up to three times more likely than the general population to die prematurely (Harden et al., 2017; Guekht et al., 2021). Their persistent episodes will lead to a decrease in quality of life (QoL), as well as injuries, depression, and social isolation (Villeneuve, 2004; Scott et al., 2017; Janson and Bainbridge, 2021). The treatment of epilepsy includes antiepileptic drugs (AEDs), surgery or ketogenic diet, and AEDs are the most commonly used treatment, which can control seizures in up to 70% of patients (Kalilani et al., 2018). However, approximately 30% of patients develop intractable epilepsy (IE), which remains a major concern in neurology that existing medications do not adequately control symptoms (Kwan et al., 2010; Moshé et al., 2015; Hansen et al., 2018). A recent review study summarized four challenges for AEDs: general side effects, economic challenges, psychological challenges, and social challenges (Mutanana et al., 2020). Severe mental, cognitive, behavioral, endocrine, and dermatological illnesses are common adverse effects of AEDs (Cai, 2017; Chen et al., 2017). Although new AEDs have been developed in recent years, the prevalence of IE has remained constant (Schmidt and Schachter, 2014). Hence, novel medications to control seizures with better efficacy and tolerability should be continually investigated to provide additional benefits for IE treatment. More and more people are seeking alternative or complementary therapies to address their ailments, and Chinese herbal medicine (CHM) is one of them.

CHM has been used for the treatment of patients with epilepsy for more than 2,000 years (Li et al., 2009). CHM therapy usually consists of a complex prescription including a number of different components with multi-target, multi-pathway synergistic regulation, among which α-asarone, β-asarone, bupleurin-α, cannabidiol, rhynchophylline, and gastrodin have been proved to have significant antiepileptic effects *in vivo* and *in vitro* experiments (Xie et al., 2013; Han et al., 2014; He Y et al., 2014; Ho et al., 2014; Liu et al., 2017; Gray and Whalley, 2020). CHM has been also widely used as an adjunct to AEDs for IE, and it has been explored to delay resistance to AEDs, thereby improving the effect of AEDs and reducing the side effects of AEDs (Wu et al., 2002; Li et al., 2012). Studies have shown that CHM may increase the concentration of AEDs in the epileptic focus by inhibiting the high expression of various drug-resistant proteins (including P-glycoprotein, multidrug resistance associated protein 1, et al.) in the brain and increasing the permeability of the blood-brain barrier to AEDs (Xie, 2011; Jia et al., 2018), or it may improve the efficacy of antiepileptic drugs by regulating the balance of excitatory and inhibitory neurotransmitters in the epileptic focus (Yan, 2014). In addition, Chinese herbal medicine has certain immunosuppressive effects, which can reduce neuroinflammation by inhibiting microglia activation and decreasing the level of pro-inflammatory cytokines, thereby reducing neuronal death and suppressing seizures (Zhang et al., 2020). However, because of the limited sample size and contradictory findings reported in these studies of the combination of CHM and AEDs for IE, it is uncertain if the present evidence is robust enough to warrant its usage. Furthermore, previous systematic reviews and meta-analyses reported flawed outcomes as inconsistent evaluation criteria were used in the included studies. Thus, to critically assess the efficacy and safety of the combination of CHM and AEDs for patients with IE, we performed this systematic review and meta-analysis.

## Methods

### Systematic Review Protocol

A protocol has been registered in PROSPERO (https://www.crd.york.ac.Uk/prospero/) for this systematic review and meta-analysis (Registration No: CRD42021290432). This systematic review and meta-analysis was conducted and reported following the Cochrane Handbook for Systematic Reviews of Interventions (Higgins and Green, 2011) and the Preferred Reporting Items for Systematic Reviews and Meta-Analyses (PRISMA) (Page et al., 2021).

### Data Sources and Search Strategy

Articles were searched in Medline, Ovid, Embase, Cochrane Library, Chinese Biomedical Database, China National Knowledge Infrastructure, VIP Database, and Wanfang Database from inception to September 2021. We used the MeSH terms in combination with free-text terms to perform a search as follows: intractable epilepsy, drug-resistant epilepsy, Chinese herbal medicine, and traditional Chinese medicine. In addition, we performed manual searches of the grey literatures and references of all included studies to avoid literature omission. The literature search was not limited to any language of publication. The search strategy was presented in Supplementary Materials Method.

### Eligibility Criteria

Inclusion Criteria: 1) Type of studies: only randomized controlled trials (RCTs) were included in this meta-analysis; 2) Type of participants: patients with age ≥18 years, diagnosed with IE (Kwan et al., 2010; China Association Against Epilepsy, 2015), regardless of age, gender, country, disease course, seizure type, and comorbidity; 3) Type of interventions: CHM combined with AEDs, regardless of dosage, treatment duration, and method of administration; 4) Type of controls: the same AEDs as intervention group, with or without the combination of placebo; 5) Type of outcomes: included the monthly seizure frequency, the abnormal rate of electroencephalogram (EEG), seizure duration, QoL, adverse events (AEs) or other clinical outcomes. The QoL was evaluated with the Quality of Life in Epilepsy-31 (QOLIE-31) questionnaire. Exclusion Criteria: 1) Quasi-randomized trials: allocation based on the date of birth and the order of admission number; 2) duplicate publications; 3) conference abstracts were excluded if the relevant data were not supplied.

### Study Selection and Data Extraction

After removing duplicates, the titles and abstracts of literatures were independently screened by two reviewers (WW Xu and Y Wang) according to the eligibility criteria. Then the full texts of the remaining literatures were downloaded and screened in detail. Any discrepancies were worked out through discussion and the addition of a third reviewer (SY Yuan).

The data were extracted by two reviewers (WW Xu and Y Wang) from the included trials independently. The detailed data included authors, title of study, year of publication, and the details of participants, intervention, controls, and outcomes. We defined the monthly seizure frequency as the primary outcome. The secondary outcomes included the abnormal rate of EEG, seizure duration, QoL, and AEs. Any undesirable or unanticipated sign, symptom, or illness, including aberrant laboratory values, was characterized as an adverse event.

### Risk of Bias Assessment

The risk of bias of included studies was assessed by two reviewers (KY Wang and LL Li) independently according to the Cochrane Handbook for Systematic Reviewers of Interventions version 5.1.0 (Higgins and Green, 2011). The items related to the methodological quality of the included RCTs were random sequence generation (selection bias), allocation concealment (selection bias), blinding of participants and personnel (performance bias), blinding of outcomes assessment (detection bias), incomplete outcomes data (attrition bias), selective reporting (reporting bias), and other biases. Each item was given a risk of bias rating of uncertain, low, or high. Any unclear items were worked out through discussion. A third reviewer (SY Yuan) would decide if necessary.

### Evidence Quality Assessment

The quality of the evidence concerning outcomes was assessed through the GRADE pro 3.6.1 program (Balshem et al., 2011; Guyatt et al., 2011). The following variables reduced the quality of each outcome: risk of bias, inconsistency, indirectness, inaccuracy, and publication bias (Guyatt et al., 2008). The evidence quality of the outcomes was rated as excellent, moderate, poor, or extremely low. Two reviewers (KY Wang and LL Li) independently assessed the quality of the included studies, and any disagreements were addressed by discussion or by a third author (SY Yuan).

### Statistical Analyses

Meta-analysis was conducted using Review Manager software (version 5.3.0; Nordic Cochrane Center, The Cochrane Collaboration, Copenhagen, Denmark). For continuous outcomes, the combined results are reported as mean difference (MD) and 95% confidence interval (CI), and the risk ratio (RR) was calculated with 95% CI for binary outcomes. The between-trials heterogeneity was measured through Cochrane’s Q test and the I^2^ statistic. I^2^ ranges from 0 to 100% based on the Cochrane Handbook for Systematic Reviews of Interventions, with values of 0%–25%, 26%–50%, 51%–75%, and 76%–100% considered insignificant, moderate heterogeneity, substantial heterogeneity, and considerable heterogeneity, respectively (Higgins and Green, 2011). Because the random-effects model assesses the outcomes of the study according to within-trial and between-trial variance, it was used to estimate the overall effect for heterogeneous studies, thus providing more conservative results (Laird and Mosteller, 1990). In addition, we used meta-regression analysis to explore the sources of heterogeneity. To assess the stability of the results, a sensitivity analysis was carried out by deleting each study one by one. Treatment duration was used to divide the participants into subgroups. The funnel plot, Begger’s test, and Egger’s test were used to discover publication bias (Egger et al., 1997).

## Results

### Literature Screening

**FIGURE 1:**
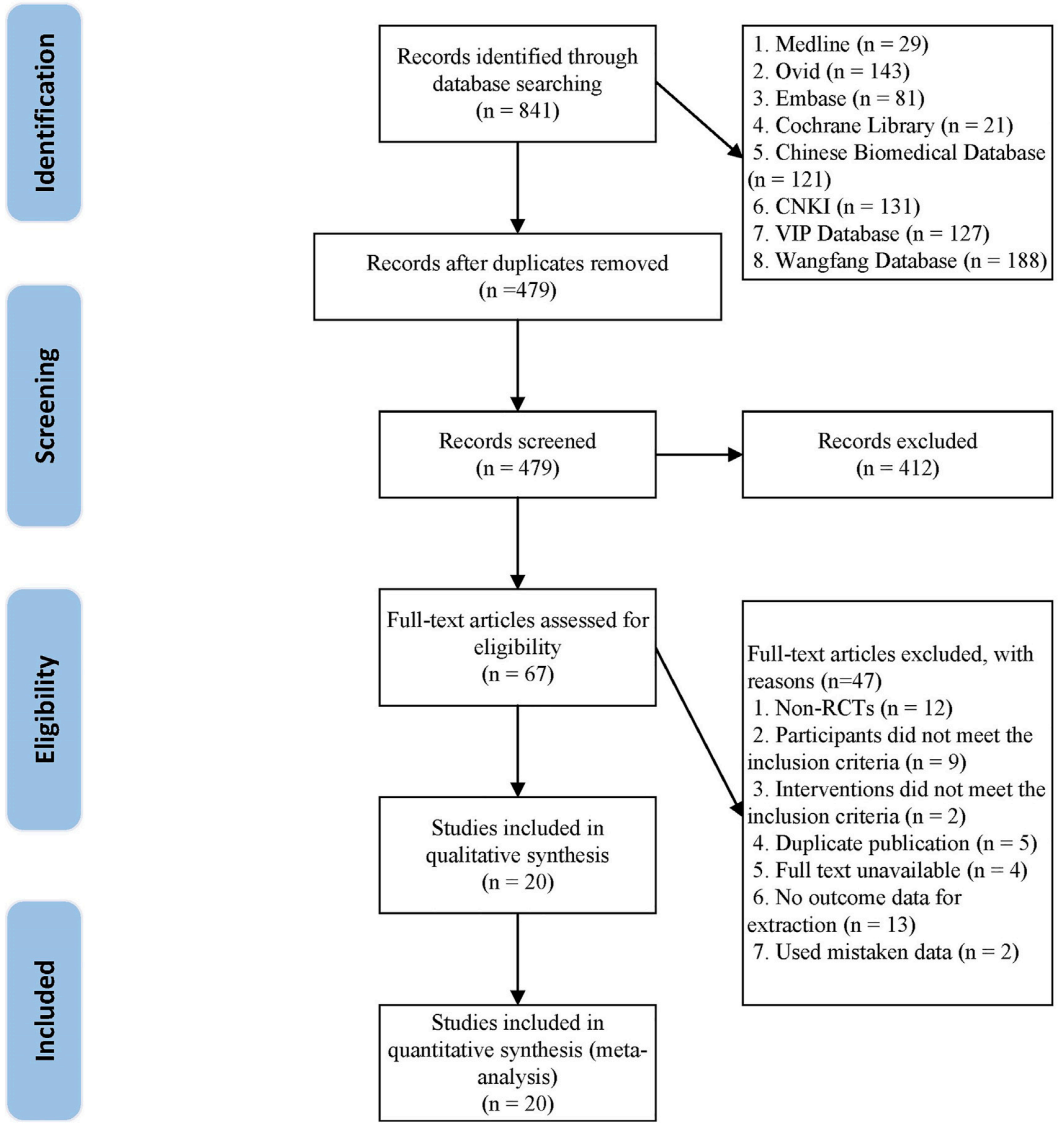
The flow chart of the study selection process.

From the initial search, a total of 841 studies were retrieved. There were 479 studies left after the duplicate studies were removed. 412 studies were eliminated after reading the title and abstract, and the remaining 67 studies were screened in detail. 47 research articles were eliminated after reading the complete text of the remaining 67 articles, as they did not match the inclusion criteria (as shown in Supplementary Table S1). Finally, 20 studies (Wen and Yan, 2010; He et al., 2011; Tian, 2011; Wang and Fan, 2012; Tang, 2013; Luo et al., 2015; Wang Q et al., 2015; Wang and Tian, 2015; Chen et al., 2016; He Q C et al., 2016; Dai and Su, 2017; Yuan and Liang, 2017; Wu, 2018; Zhang and Guo, 2018; Chen et al., 2019; Shang, 2019; Xie et al., 2019; Zhang, 2019; Liu et al., 2020; Liu, 2021) were eligible and included in the meta-analysis. The flowchart of study screening is shown in Figure 1.

### Characteristics of Included Studies

A total of 1,830 patients (CHM+AEDs group: 951 patients; AEDs group: 879 patients) with IE from 20 studies were included in this meta-analysis. All the studies were performed in China, and the published times ranged from 2010 to 2021. Only three of the included studies used a multicenter design (He et al., 2011; Tian, 2011; Chen et al., 2016), and the others were single-center trials. Most studies recruited more male patients. Patients with IE after a stroke were included in one trial (Liu et al., 2020), and patients with IE after brain trauma were included in another (Wang and Tian, 2015). The remaining RCTs simply showed that patients with IE were included.

The treatment duration lasted from 4 to 24 weeks, and most of them lasted 12 weeks (7/20, 35%). Except for one study (He et al., 2011), which used AEDs + placebo in the control group, the remaining studies compared the efficacy of CHM+AEDs (experiment group) and AEDs (control group) in the treatment of IE. All included studies used oral CHM preparations and formulation forms varied: 1 study (Wang and Fan, 2012) used capsule, 3 granules (Wen and Yan, 2010; Tian, 2011; Wang Q et al., 2015), 1 tablet (He et al., 2011), 2 pills (Luo et al., 2015; Chen et al., 2019), and 13 formula decoctions (Tang, 2013; Wang and Tian, 2015; Chen et al., 2016; He Q C et al., 2016; Dai and Su, 2017; Yuan and Liang, 2017; Wu, 2018; Zhang and Guo, 2018; Shang, 2019; Xie et al., 2019; Zhang, 2019; Liu et al., 2020; Liu, 2021).

In terms of the treatment measures in the control group, one research (Liu et al., 2020) used oxcarbazepine, one research (Wang and Tian, 2015) used sodium valproate, one research used sodium valproate combined with lamotrigine (Chen et al., 2019), six studies used carbamazepine combined with clonazepam (Tang, 2013; Wang Q et al., 2015; Yuan and Liang, 2017; Wu, 2018; Zhang and Guo, 2018; Zhang, 2019), and the remaining trials did not describe the prescription of AEDs in detail. In terms of the outcomes, the monthly seizure frequency was reported in 12 studies (Wang and Fan, 2012; Tang, 2013; Luo et al., 2015; Wang Q et al., 2015; Wang and Tian, 2015; Yuan and Liang, 2017; Wu, 2018; Zhang and Guo, 2018; Chen et al., 2019; Xie et al., 2019; Zhang, 2019; Liu et al., 2020), the abnormal rate of EEG in 11 (Wen and Yan, 2010; Tian, 2011; Wang and Fan, 2012; Tang, 2013; Chen et al., 2016; He Q C et al., 2016; Yuan and Liang, 2017; Zhang and Guo, 2018; Shang, 2019; Xie et al., 2019; Zhang, 2019), the seizure duration in 3 (Chen et al., 2019; Liu et al., 2020; Liu, 2021), the QoL in 4 (Tian, 2011; Wu, 2018; Shang, 2019; Xie et al., 2019), and the AEs in 11 (Wen and Yan, 2010; He et al., 2011; Tian, 2011; Wang and Fan, 2012; Luo et al., 2015; Wang Q et al., 2015; Dai and Su, 2017; Wu, 2018; Xie et al., 2019; Liu et al., 2020; Liu, 2021). The characteristics of the included studies are summarized in Table 1, and the composition and dosage of CHMt reported by the included trials are summarized in Table 2.

**TABLE 1:** | Characteristics of the included RCTs and the detail of PICOS.

Study ID	Sample size (T/C)		Age (Y)		Gender (M/F)		Course of disease (Y)		Intervention		Duration (week)	Out-comes	Jadad score
		T		C	T	C	T	C	T	C			
Liu (2021)	30/30	49.22 ± 14.61		49.03 ± 14.43	14/16	15/15	5.63 ± 2.51	5.66 ± 2.77	Chaibeizhixian decoction + control	Conventional AEDs	12	③⑤	3
Liu et al. (2020)	48/48	76.03 ± 10.01		75.21 ± 9.36	22/26	21/27	8.02 ± 2.96	7.42 ± 2.51	Kangxianjiejing decoction + control	Conventional treatment + oxcarbazepine	24	①③⑤	1
Chen et al. (2019)	32/32	58.20 ± 25.60		60.00 ± 26.90	12/20	16/16	8.40 ± 3.20	8.80 ± 3.90	Dingxian pill + control	sodium valproate sustained-release tablet + lamotrigine	24	①③	1
Shang (2019)	50/50	34.93 ± 5.22		34.72 ± 5.71	26/24	30/20	4.38 ± 1.34	4.57 ± 1.51	Zhebeishuganzhixian formula + control	Multiple AEDs (carbamazepine, VPA, levetiracetam)	16	②④⑤	1
Xie et al. (2019)	44/43	25.66 ± 5.95		26.53 ± 6.32	NA	NA	6.52 ± 4.01	6.74 ± 3.93	Qingxinwendan decoction + control	Conventional AEDs	24	①②④⑤	3
Zhang (2019)	40/40	44.60 ± 5.70		44.80 ± 6.20	18/22	19/21	2.50 ± 0.60	2.30 ± 0.80	Self-made formula + Control	Carbamazepine/+ clonazepam	8	①②	1
Wu (2018)	41/41	42.70 ± 10.10		42.50 ± 10.70	23/18	22/19	NA	NA	Self-made formula + control	Carbamazepine/+ clonazepam	8	①④⑤	1
Zhang and Guo (2018)	43/43	46.28 ± 8.12		45.85 ± 7.96	26/17	27/16	3.14 ± 0.83	3.25 ± 0.79	Self-made formula + control	Carbamazepine/+ clonazepam	12	①②	1
Dai and Su (2017)	45/45	32.35 ± 3.11		32.26 ± 3.07	29/16	27/18	26.26 ± 3.38	26.19 ± 3.28	Chaibeizhixian decoction + control	Multiple AEDs (carbamazepine, VPA, levetiracetam, etc.)	24	⑤	2
Yuan and Liang (2017)	50/50	42.60 ± 2.20		41.40 ± 2.50	24/26	22/28	4.90 ± 0.40	4.40 ± 0.20	Self-made formula + control	Carbamazepine/+ clonazepam	8	①②	2
He Q C et al. (2016)	60/60	40.47 ± 3.46		39.53 ± 3.62	26/34	23/37	4.25 ± 1.83	4.43 ± 1.58	Chaihushugan decoction + control	Multiple AEDs (carbamazepine, VPA, levetiracetam)	16	②	2
Chen et al. (2016)	30/31	31.70 ± 13.2		31.90 ± 11.90	17/13	15/16	NA	NA	Qutandingxian decoction + control	Conventional AEDs	12	②	1
Luo et al. (2015)	66/66	42.50 ± 11.40		43.20 ± 12.20	38/28	39/27	NA	NA	Annao pill + control	Multiple AEDs (carbamazepine, VPA, levetiracetam, etc.)	4	①⑤	2
Wang Q et al. (2015)	20/20	41.20 ± 12.8		42.50 ± 11.20	11/9	12/8	NA	NA	Pingganzhixian granule + control	Carbamazepine/+ clonazepam	4	①⑤	2
Wang and Tian (2015)	40/40	64.20 ± 4.40		63.90 ± 4.60	26/14	28/12	2.40 ± 0.40	2.70 ± 0.30	Xuefuzhuyu decoction + control	VPA	12	①	2
Tang (2013)	61/61	42.50 ± 11.30		44.70 ± 15.60	36/25	34/27	2.40 ± 0.60	2.30 ± 0.90	Self-made formula + control	Carbamazepine/+ clonazepam	8	①②	1
Wang and Fan (2012)	20/16	43.50		51.20	11/9	9/7	7.30	6.20	Huangzhudingxian capsule + control	VPA/carbamazepine	16	①②⑤	1
He et al. (2011)	137/69	33.99 ± 15.05		33.90 ± 16.30	87/50	41/28	NA	NA	Dianxianning pill + control	Conventional AEDs + placebo	12	⑤	5
Tian (2011)	31/31	30.68 ± 1.94		29.71 ± 1.66	22/9	20/11	10.81 ± 1.28	10.91 ± 0.99	Yuxianling granule + control	Conventional AEDs	12	②④⑤	3
Wen and Yan (2010)	63/63	49.00		51.00	40/23	39/24	6.50	8.50	Dingxian granule + control	Conventional AEDs	12	②⑤	1

T, treatment group; C, control group; Y, year; M: male; F, female; NA, not applicable; AEDs, antiepileptic drugs; VPA, valproic acid; Outcomes: ①, monthly seizure frequency; ②, abnormal rate of electroencephalogram; ③, seizure duration; ④, QOL-31 quality of life scale scores; ⑤, adverse events.

**TABLE 2:** The composition and dosage of CHM reported in each trial.

First author (publication year)	The composition and dosage of CHM	Frequency of administration
Liu (2021)	*Bupleurum Chinense* DC. [*Apiaceae*; Bupleuri radix] 12 g	Standardized CHM, 200 ml bid
	*Gastrodia elata Blume* [*Orchidaceae*; Gastrodiae rhizoma] 15 g	
	*Fritillaria thunbergii* Miq. [*Liliaceae*; *Fritillariae thunbergii* bulbus] 9 g	
	*Pinellia ternata* (Thunb.) Makino [*Araceae*; Pinelliae rhizoma praeparatum] 9 g	
	*Acorus calamus* var. angustatus Besser [*Acoraceae*; Acori tatarinowii rhizoma]9 g	
	*Ostrea gigas* Thunberg [*Ostreidae*; Ostreae concha] 30 g	
	*Pheretima aspergillum* (E. Perrier) [*Megascolecidae*; Pheretima] 6 g	
	*Bombyx mori* Linnaeus [Silkworm pilgrimaging; Bombyx batryticatus] 6 g	
	*Setaria italica* (L.) P.Beauv. [*Poaceae*; Setariae fructus germinatus] 10 g	
	*Hordeum vulgare* L. [*Poaceae*; Hordei fructus germinatus] 10 g	
Liu et al. (2020)	Magnetitum 30 g	Standardized or individualized CHM, 300 ml bid
	*Cyathula officinalis* K.C.Kuan [*Amaranthaceae*; Cyathulae radix] 30 g	
	*Poria cocos* (Schw.)Wolf [*Polyporaceae*; Poria] 30 g	
	*Arisaema erubescens* (Wall.) Schott [*Araceae*; Arisaema cum bile] 15 g	
	*P. aspergillum* (E. Perrier) [*Megascolecidae*; *Pheretima*] 15 g	
	*P. ternata* (Thunb.) Makino [*Araceae*; Pinelliae rhizoma praeparatum cum alumine] 12 g	
	*B. mori* Linnaeus [Silkworm pilgrimaging; Bombyx batryticatus] 10 g	
	*Curcuma longa* L. [*Zingiberaceae*; Curcumae longae rhizoma] 10 g	
	*Rheum palmatum* L. [*Polygonaceae*; Rhei radix et rhizoma] 10 g	
	*Citrus × aurantium* L. [Rutaceae; Citri exocarpium rubrum] 10 g	
	*A. calamus* var. angustatus Besser [*Acoraceae*; Acori tatarinowii rhizoma] 10 g	
	*Ligusticum* chuanxiong Hort. [*Apiaceae*; Chuanxiong rhizoma] 10 g	
	*Neolitsea cassia* (L.) Kosterm. [*Lauraceae*; Cinnamomi ramulus] 10 g	
	*Panax ginseng* C.A.Mey. [*Araliaceae*; Ginseng radix et rhizoma] 10 g	
	*Glycyrrhiza uralensis Fisch*. ex DC. [*Fabaceae*; Glycyrrhizae radix et rhizoma] 10 g	
	*Cryptotympana pustulata* Fabricius [Cicadidae; Cicadae periostracum] 6 g	
	*Aquilaria sinensis* (Lour.) Spreng. [Thymelaeaceae; Aquilariae lignum resinatum] 6 g	
Chen et al. (2019)	*Fritillaria cirrhosa* D.Don [*Liliaceae*; Fritillariae cirrhosae bulbus] 6 g	Standardized CHM, 6 g bid
	*B. mori* Linnaeus [ Silkworm pilgrimaging; Bombyx batryticatus] 3 g	
	*C. × aurantium* L. [Rutaceae; Citri reticulatae pericarpium] 4 g	
	*A. erubescens* (Wall.) Schott [*Araceae*; Arisaema cum bile] 3 g	
	Salvia miltiorrhiza Bunge [*Lamiaceae*; Salviae miltiorrhizae radix et rhizoma] 12 g	
	*Juncus effusus* L. [Juncaceae; Junci medulla] 3 g	
	*P. cocos* (Schw.)Wolf [*Polyporaceae*; Poria] 6 g	
	*G. elata* Blume [*Orchidaceae*; Gastrodiae rhizoma] 6 g	
	Succinum 6 g	
	*P. ternata* (Thunb.) Makino [*Araceae*; Pinelliae rhizoma praeparatum cum zingibere et alumine] 6 g	
	Buthus martensii Karsch [*Buthidae*; Scorpio] 3 g	
	*A. calamus* var. angustatus Besser [*Acoraceae*; Acori tatarinowii rhizoma] 3 g	
	*Zingiber officinale Roscoe* [*Zingiberaceae*; Zingiberis rhizoma recens] 9 g	
	*Ophiopogon japonicus* (Thunb.) Ker Gawl. [*Asparagaceae*; Ophiopogonis radix] 12 g	
	*P. ginseng* C.A.Mey. [*Araliaceae*; Ginseng radix et rhizoma] 3 g	
	*G. uralensis* Fisch. ex DC. [*Fabaceae*; Glycyrrhizae radix et rhizoma] 3 g	
	Cinnabaris 2 g	
Shang (2019)	*F. thunbergii* Miq. [*Liliaceae*; Fritillariae thunbergii bulbus] 15 g	Standardized or individualized CHM, 1 package bid
	*G. elata* Blume [*Orchidaceae*; Gastrodiae rhizoma] 15 g	
	*Bupleurum Chinense* DC. [*Apiaceae*; Bupleuri radix] 10 g	
	*L. chuanxiong* Hort. [*Apiaceae*; Chuanxiong rhizoma] 10 g	
	*Cyperus* rotundus L. [*Cyperaceae*; Cyperi rhizoma] 10 g	
	*P. lactiflora* Pall. [*Paeoniaceae*; Paeoniae radix alba] 10 g	
	*C. × aurantium* L. [*Rutaceae*; Citri reticulatae pericarpium] 10 g	
	*A. calamus* var. angustatus Besser [*Acoraceae*; Acori tatarinowii rhizoma] 10 g	
	*P. aspergillum* (E. Perrier) [*Megascolecidae*; Pheretima]6 g	
	*G. uralensis Fisch*. ex DC. [Fabaceae; Glycyrrhizae radix et rhizoma praeparata cum melle] 6 g	
Xie et al. (2019)	*C. × aurantium* L. [Rutaceae; Citri reticulatae pericarpium] 10 g	Standardized CHM, 125 ml bid
	*P. ternata* (Thunb.) Makino [*Araceae*; Pinelliae rhizoma praeparatum] 10 g	
	*P. cocos* (Schw.)Wolf [*Polyporaceae*; Poria] 10 g	
	*C. × aurantium* L. [*Rutaceae*; Aurantii fructus immaturus] 10 g	
	*B. tuldoides* Munro [*Poaceae*; Bambusae caulis in taenias] 10 g	
	Atractylodes macrocephala Koidz. [*Asteraceae*; Atractylodis macrocephalae rhizoma] 10 g	
	*A. calamus* var. angustatus Besser [*Acoraceae*; Acori tatarinowii rhizoma] 10 g	
	*Coptis Chinensis* Franch. [*Ranunculaceae*; Coptidis rhizoma] 5 g	
	*P. lactiflora* Pall. [*Paeoniaceae*; Paeoniae radix alba] 10 g	
	*Angelica sinensis* (Oliv.) Diels [*Apiaceae*; Angelicae sinensis radix] 10 g	
	*C. rotundus* L. [*Cyperaceae*; Cyperi rhizoma] 10 g	
	*O. japonicus* (Thunb.) Ker Gawl. [*Asparagaceae*; Ophiopogonis radix] 8 g	
	*L. chuanxiong* Hort. [*Apiaceae*; Chuanxiong rhizoma] 6 g	
	*Polygala tenuifolia* Willd. [*Polygalaceae*; Polygalae radix] 6 g	
	*P ginseng* C.A.Mey. [*Araliaceae*; Ginseng radix et rhizoma] 6 g	
	*G. uralensis* Fisch. ex DC. [Fabaceae; Glycyrrhizae radix et rhizoma] 4 g	
Zhang (2019)	*P. cocos* (Schw.)Wolf [*Polyporaceae*; Poria] 20 g	Standardized or individualized CHM, 50 ml bid
	*P. aspergillum* (E. Perrier) [*Megascolecidae*; Pheretima] 20 g	
	Whitmania pigra Whitman [Hirudinidae; Hirudo] 10 g	
	*B. tuldoides* Munro [*Poaceae*; Bambusae caulis in taenias] 10 g	
	*C. × aurantium* L. [Rutaceae; Citri exocarpium rubrum] 10 g	
	*P. ternata* (Thunb.) Makino [*Araceae*; Pinelliae rhizoma praeparatum] 10 g	
	*G. uralensis* Fisch. ex DC. [Fabaceae; Glycyrrhizae radix et rhizoma] 5 g	
	Buthus martensii Karsch [Buthidae; Scorpio] 5 g	
Wu (2018)	*P. cocos* (Schw.)Wolf [*Polyporaceae*; Poria] 5 g	Standardized or individualized CHM, 50 ml bid
	*C. × aurantium* L. [Rutaceae; Citri exocarpium rubrum] 1 g	
	*Bambusa tuldoides* Munro [*Poaceae*; Bambusae caulis in taenias] 8 g	
	*A. calamus* var. angustatus Besser [*Acoraceae*; Acori tatarinowii rhizoma] 8 g	
	*P. aspergillum* (E. Perrier) [*Megascolecidae*; Pheretima] 15 g	
	*G. uralensis* Fisch. ex DC. [Fabaceae; Glycyrrhizae radix et rhizoma] 8 g	
	*P. ternata* (Thunb.) Makino [*Araceae*; Pinelliae rhizoma praeparatum] 8 g	
	Whitmania pigra Whitman [Hirudinidae; Hirudo] 8 g	
	Buthus martensii Karsch [Buthidae; Scorpio] 3 g	
	*Dioscorea oppositifolia* L. [Dioscoreaceae; Dioscoreae rhizoma] 10 g	
	*P. tenuifolia* Willd. [Polygalaceae; Polygalae radix] 10 g	
Zhang and Guo (2018)	*P. cocos* (Schw.)Wolf [*Polyporaceae*; Poria] 20 g	Standardized or individualized CHM, 50 ml bid
	*P. aspergillum* (E. Perrier) [*Megascolecidae*; Pheretima] 20 g	
	*C. × aurantium* L. [Rutaceae; Citri exocarpium rubrum] 15 g	
	Whitmania pigra Whitman [Hirudinidae; Hirudo] 10 g	
	*B. tuldoides* Munro [*Poaceae*; Bambusae caulis in taenias] 10 g	
	*A. calamus* var. angustatus Besser [*Acoraceae*; Acori tatarinowii rhizoma] 10 g	
	*P.ternata* (Thunb.) Makino [*Araceae*; Pinelliae rhizoma] 10 g	
	*G. uralensis* Fisch. ex DC. [*Fabaceae*; Glycyrrhizae radix et rhizoma] 5 g	
	Buthus martensii Karsch [Buthidae; Scorpio] 5 g	
Dai and Su (2017)	*F. thunbergii* Miq. [*Liliaceae*; Fritillariae thunbergii bulbus] 20 g	Standardized or individualized CHM, 1 package bid
	*B. Chinense* DC. [*Apiaceae*; Bupleuri radix] 20 g	
	*O.* gigas Thunberg [Ostreidae; Ostreae concha] 15 g	
	*A. calamus* var. angustatus Besser [*Acoraceae*; Acori tatarinowii rhizoma] 15 g	
	*G. elata* Blume [*Orchidaceae*; Gastrodiae rhizoma] 15 g	
	*P. aspergillum* (E. Perrier) [*Megascolecidae*; Pheretima] 8 g	
Yuan and Liang (2017)	*P. cocos* (Schw.)Wolf [*Polyporaceae*; Poria] 20 g	Standardized or individualized CHM, 50 ml bid
	*P. aspergillum* (E. Perrier) [*Megascolecidae*; Pheretima] 20 g	
	*C. × aurantium* L. [Rutaceae; Citri exocarpium rubrum] 15 g	
	*A. calamus* var. angustatus Besser [*Acoraceae*; Acori tatarinowii rhizoma] 10 g	
	*P. ternata* (Thunb.) Makino [*Araceae*; Pinelliae rhizoma] 10 g	
	*B. tuldoides* Munro [*Poaceae*; Bambusae caulis in taenias] 10 g	
	Whitmania pigra Whitman [Hirudinidae; Hirudo] 10 g	
	*G. uralensis* Fisch. ex DC. [Fabaceae; Glycyrrhizae radix et rhizoma] 5 g	
	Buthus martensii Karsch [Buthidae; Scorpio] 5 g	
He Q C et al. (2016)	*B. Chinense* DC. [*Apiaceae*; Bupleuri radix] 12 g	Standardized or individualized CHM, 1 package bid
	*C. × aurantium* L. [Rutaceae; Citri reticulatae pericarpium] 12 g	
	Ligus chuanxiong Hort. [*Apiaceae*; Chuanxiong rhizoma] 9 g	
	*C. × aurantium* L. [*Rutaceae*; Aurantii fructus] 9 g	
	*P. lactiflora* Pall. [*Paeoniaceae*; Paeoniae radix alba] 9 g	
	*C rotundus* L. [*Cyperaceae*; Cyperi rhizoma] 9 g	
	*G. uralensis* Fisch. ex DC. [*Fabaceae*; Glycyrrhizae radix et rhizoma praeparata cum melle] 6 g	
	*F. thunbergii* Miq. [*Liliaceae*; Fritillariae thunbergii bulbus] 12 g	
Chen et al. (2016)	*A. erubescens* (Wall.) Schott [*Araceae*; Arisaematis rhizoma preparatum] 10 g	Standardized or individualized CHM, 1 package bid
	*P. ternata* (Thunb.) Makino [*Araceae*; Pinelliae rhizoma] 9 g	
	*C. × aurantium* L. [Rutaceae; Citri reticulatae pericarpium] 9 g	
	*P. cocos* (Schw.)Wolf [*Polyporaceae*; Poria] 9 g	
	*G. elata* Blume [*Orchidaceae*; Gastrodiae rhizoma] 10 g	
	*Uncaria rhynchophylla* (Miq.) Miq. [*Rubiaceae*; Uncariae ramulus cum uncis] 15 g	
	*Scolopendra subspinipes mutilans* L. Koch [Scolopendridae; Scolopendra] 1 strip	
	Sa miltiorrhiza Bunge [*Lamiaceae*; Salviae miltiorrhizae radix et rhizoma] 18 g	
	*A. calamus* var. angustatus Besser [*Acoraceae*; Acori tatarinowii rhizoma] 12 g	
	*P. tenuifolia* Willd. [*Polygalaceae*; Polygalae radix] 10 g	
	*L. chuanxiong* Hort. [*Apiaceae*; Chuanxiong rhizoma] 12 g	
	*Z. officinale* Roscoe [*Zingiberaceae*; Zingiberis rhizoma recens] 4 slices	
	Ziziphus jujuba Mill. [*Rhamnaceae*; Jujubae fructus] 9 g	
Luo et al. (2015)	*Bos taurus* domesticus Gemlin [Bovidae; Bovis calculus]	Standardized CHM, 6 g bid
	*Sus scrofa* domestica Brisson [Suis fellis pulvis]	
	Cinnabaris	
	Dryobalanops aromatica C.F.Gaertn. [Dipterocarpaceae; Borneolum syntheticum]	
	Bubalus bubalis Linnaeus [Bovidae; Cornu bubali]	
	*P. martensii* (Dunker) [Pteriidae; Margarita]	
	Scutellaria baicalensis Georgi [*Lamiaceae*; Scutellariae radix]	
	*Coptis Chinensis* Franch. [Ranunculaceae; Coptidis rhizoma]	
	Gardenia jasminoides J.Ellis [Rubiaceae; Gardeniae fructus]	
	Realgar	
	*Curcuma aromatica* Salisb. [*Zingiberaceae*; Curcumae radix]	
	Gypsum fibrosum	
	Haematitum	
	Pteria martensii (Dunker) [Pteriidae; Margarita] l-menthol	
Wang Q et al. (2015)	Gastrodia elata Blume [*Orchidaceae*; Gastrodiae rhizoma] 20 g	Standardized CHM, 125 ml bid
	Buthus martensii Karsch [Buthidae; Scorpio] 6 g	
	*A. erubescens* (Wall.) Schott [*Araceae*; Arisaema cum bile] 9 g	
	*C. aromatica* Salisb. [*Zingiberaceae*; Curcumae radix] 20 g	
	*A. calamus* var. angustatus Besser [*Acoraceae*; Acori tatarinowii rhizoma] 12 g	
Wang and Tian (2015)	Os Draconis 20 g	Standardized or individualized CHM, 250 ml bid
	*U. rhynchophylla* (Miq.) Miq. [*Rubiaceae*; Uncariae ramulus cum uncis] 15 g	
	*Prunus persica* (L.) Batsch [*Rosaceae*; Persicaese men] 12 g	
	*G. elata* Blume [*Orchidaceae*; Gastrodiae rhizoma] 10 g	
	*A. erubescens* (Wall.) Schott [*Araceae*; Arisaema cum bile] 10 g	
	*A. sinensis* (Oliv.) Diels [*Apiaceae*; Angelicae sinensis radix] 9 g	
	*C. officinalis* K.C.Kuan [*Amaranthaceae*; Cyathulae radix] 9 g	
	*Rehmannia glutinosa* (Gaertn.) DC. [Orobanchaceae; Rehmanniae radix] 9 g	
	*C. tinctorius* L. [*Asteraceae;* Carthami flos] 9 g	
	*C. × aurantium* L. [*Rutaceae*; Aurantii fructus] 6 g	
	*P. anomala* subsp. veitchii (Lynch) D.Y.Hong and K.Y.Pan [*Paeoniaceae*; Paeoniae radix rubra] 6 g	
	*B. Chinense* DC. [*Apiaceae*; Bupleuri radix] 5 g	
	*Ligusticum* chuanxiong Hort. [*Apiaceae*; Chuanxiong rhizoma] 5 g	
	*Platycodon grandiflorus* (Jacq.) A.DC. [Campanulaceae; Platycodonis radix] 5 g	
	Buthus martensii Karsch [Buthidae; Scorpio] 3 g	
	*G. uralensis* Fisch. ex DC. [Fabaceae; Glycyrrhizae radix et rhizoma] 3 g	
Tang (2013)	*P. cocos* (Schw.)Wolf [*Polyporaceae*; Poria] 20 g	Standardized or individualized CHM, 50 ml bid
	*C. × aurantium L*. [*Rutaceae*; Citri exocarpium rubrum] 15 g	
	*B. tuldoides* Munro [*Poaceae*; Bambusae caulis in taenias] 10 g	
	*A. calamus* var. angustatus Besser [*Acoraceae*; Acori tatarinowii rhizoma] 10 g	
	*P. aspergillum* (E. Perrier) [*Megascolecidae*; Pheretima] 20 g	
	*G. uralensis* Fisch. ex DC. [Fabaceae; Glycyrrhizae radix et rhizoma] 5 g	
	Whitmania pigra Whitman [Hirudinidae; Hirudo] 10 g	
	Buthus martensii Karsch [Buthidae; Scorpio] 5 g	
	*P. ternata* (Thunb.) Makino [*Araceae*; Pinelliae rhizoma] 10 g	
Wang and Fan (2012)	*C. chinensis* Franch. [*Ranunculaceae*; Coptidis rhizoma]	Standardized CHM, 3 capsules tid
	*B. tuldoides* Munro [*Poaceae*; Bambusae caulis in taenias]	
	*C. × aurantium* L. [*Rutaceae*; Aurantii fructus immaturus]	
	*B. Chinense* DC. [*Apiaceae*; Bupleuri radix]	
	Os Draconis	
	*O.* gigas Thunberg [*Ostreidae*; Ostreae concha]	
	*C. aromatica* Salisb. [*Zingiberaceae*; Curcumae radix]	
	*B. mori* Linnaeus [ Silkworm pilgrimaging; Bombyx batryticatus]	
He et al. (2011)	Valeriana jatamansi Jones ex Roxb. [Caprifoliaceae; Valerianae jatamansi rhizoma et radix]	Standardized CHM, 4 pills tid
	*A. calamus* var. angustatus Besser [*Acoraceae*; Acori tatarinowii rhizoma]	
	*U. rhynchophylla* (Miq.) Miq. [*Rubiaceae*; Uncariae ramulus cum uncis]	
	Ipomoea nil (L.) Roth [Convolvulaceae; Pharbitidis semen]	
	*Euphorbia lathyri*s L. [Euphorbiaceae; Euphorbiae semen]	
	*Valeriana officinalis* L. [Valerianaceae; Valerianae jatamansi rhizoma et radix]	
	Nardostachys jatamansi (D.Don) DC. [Caprifoliaceae; Nardostachyos radix et rhizoma] l-menthol	
Tian (2011)	Ligus chuanxiong Hort. [*Apiaceae*; Chuanxiong rhizoma] 10 g	Standardized CHM, 8 g bid
	*A. calamus* var. angustatus Besser [*Acoraceae*; Acori tatarinowii rhizoma] 10 g	
	*C. tinctorius* L. [Asteraceae; Carthami flos] 5 g	
	*B. tuldoides* Munro [*Poaceae*; Bambusae caulis in taenias] 10 g	
	Micae lapis aureus 3 g	
	Selenarctos thibetanus G. Cuvier [Ursidae; Ursi fellis pulvis] 0.1 g	
	Scutellaria baicalensis Georgi [*Lamiaceae*; Scutellariae radix] 6 g	
	Buthus martensii Karsch [Buthidae; Scorpio] 3 g	
	*S. subspinipes* mutilans L. Koch [Scolopendridae; Scolopendra] 2 strips	
	*B. mori* Linnaeus [ Silkworm pilgrimaging; Bombyx batryticatus] 10 g	
	*P. aspergillum* (E. Perrier) [*Megascolecidae*; Pheretima] 10 g	
	*Eleutherococcus senticosus* (Rupr. and Maxim.) Maxim. [Araliaceae; Acanthopanacis senticosi radix et rhizoma seu caulis] 15 g	
	*Dryobalanops aromatica* C.F.Gaertn. [Dipterocarpaceae; Borneolum syntheticum] 0.1 g	
Wen and Yan (2010)	*B. mori* Linnaeus [ Silkworm pilgrimaging; Bombyx batryticatus] 10 g	Standardized CHM, 1.5 g tid
	*P. aspergillum* (E. Perrier) [*Megascolecidae*; Pheretima] 10 g	
	Dens draconis 10 g	
	*P. cocos* (Schw.)Wolf [*Polyporaceae*; Poria] 10 g	
	*A. sinensis* (Oliv.) Diels [*Apiaceae*; Angelicae sinensis radix] 10 g	
	*P. lactiflora* Pall. [*Paeoniaceae*; Paeoniae radix alba] 10 g	
	*G. uralensis* Fisch. ex DC. [Fabaceae; Glycyrrhizae radix et rhizoma praeparata cum melle] 10 g	

CHM, Chinese herbal medicine; ml, milliliter; g, gram; bid, twice a day; tid, three times a day.

### Risk of Bias in Studies

**FIGURE 2:**
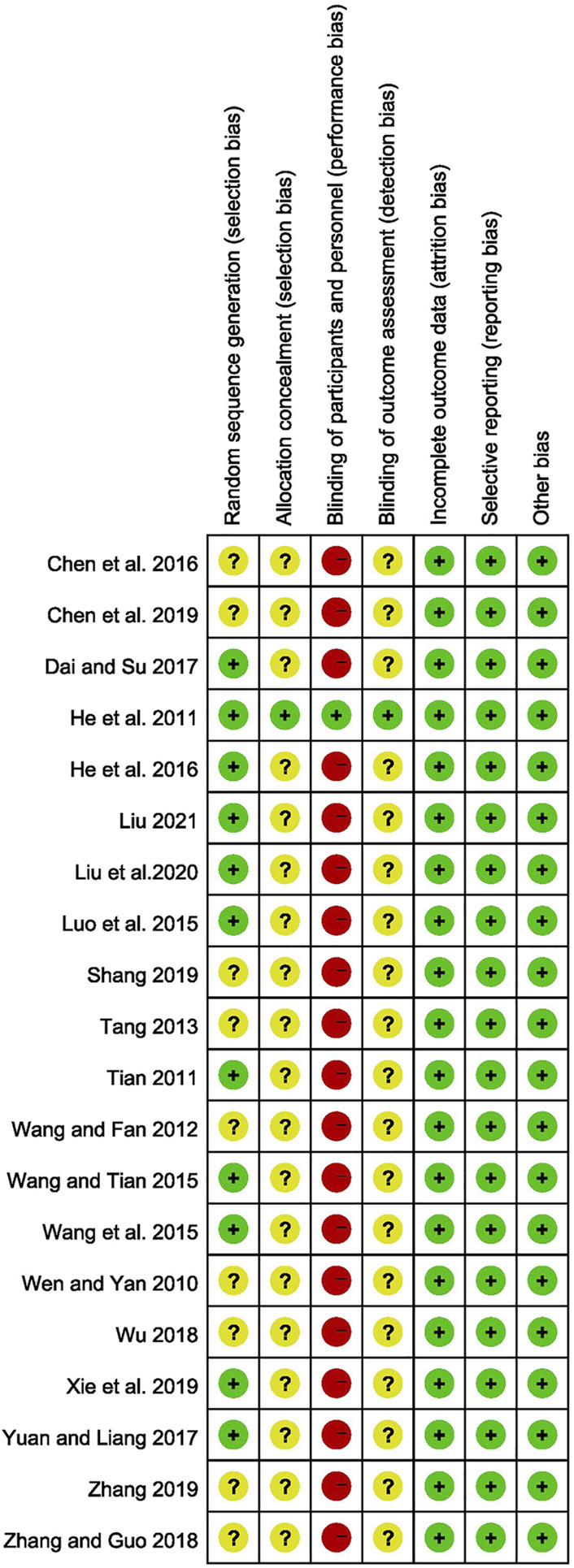
Risk of bias summary.

All of the studies mentioned randomization. 11 of them specifically mentioned randomization methods, such as random number tables (Luo et al., 2015; Wang Q et al., 2015; Wang and Tian, 2015; He Q C et al., 2016; Dai and Su, 2017; Yuan and Liang, 2017; Xie et al., 2019; Liu et al., 2020; Liu, 2021), central randomization system (He et al., 2011), and DOLL’s clinical randomization table (Tian, 2011), which were considered to have a low risk of bias, and the risk of bias for the remaining studies were unclear. Except for one study (He et al., 2011), none of the other studies described the allocation concealment and blind approach, which were considered to have a high risk of bias in the blinding of participants and personnel. Except for 1 study (He et al., 2011), all other studies were unclear on the blinding of outcome assessment. Four trials (He et al., 2011; Tian, 2011; Xie et al., 2019; Liu, 2021) provided the number of dropouts and the reasons for them, whereas the remaining studies had no missing data on outcomes. As a result, all of the included studies were rated as having a low risk of attrition bias. All studies have a low-risk selective reporting bias. All included studies were comparable at baseline and considered low risk. The Risk of bias of included studies were summarized in Figure 2.

## Overall Results of Meta-Analysis

### Monthly Seizure Frequency

**FIGURE 3:**
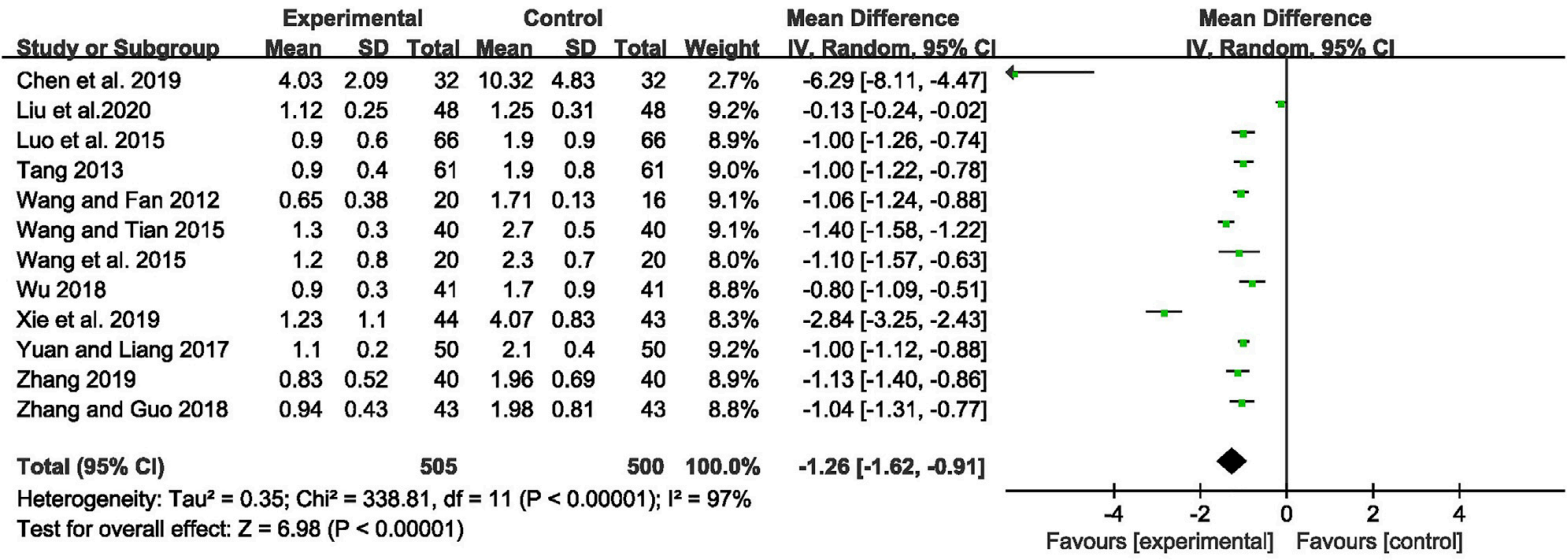
Forest plot displaying the effects of CHM+AEDs vs. AEDs for monthly seizure frequency in patients with IE.

**FIGURE 4:**
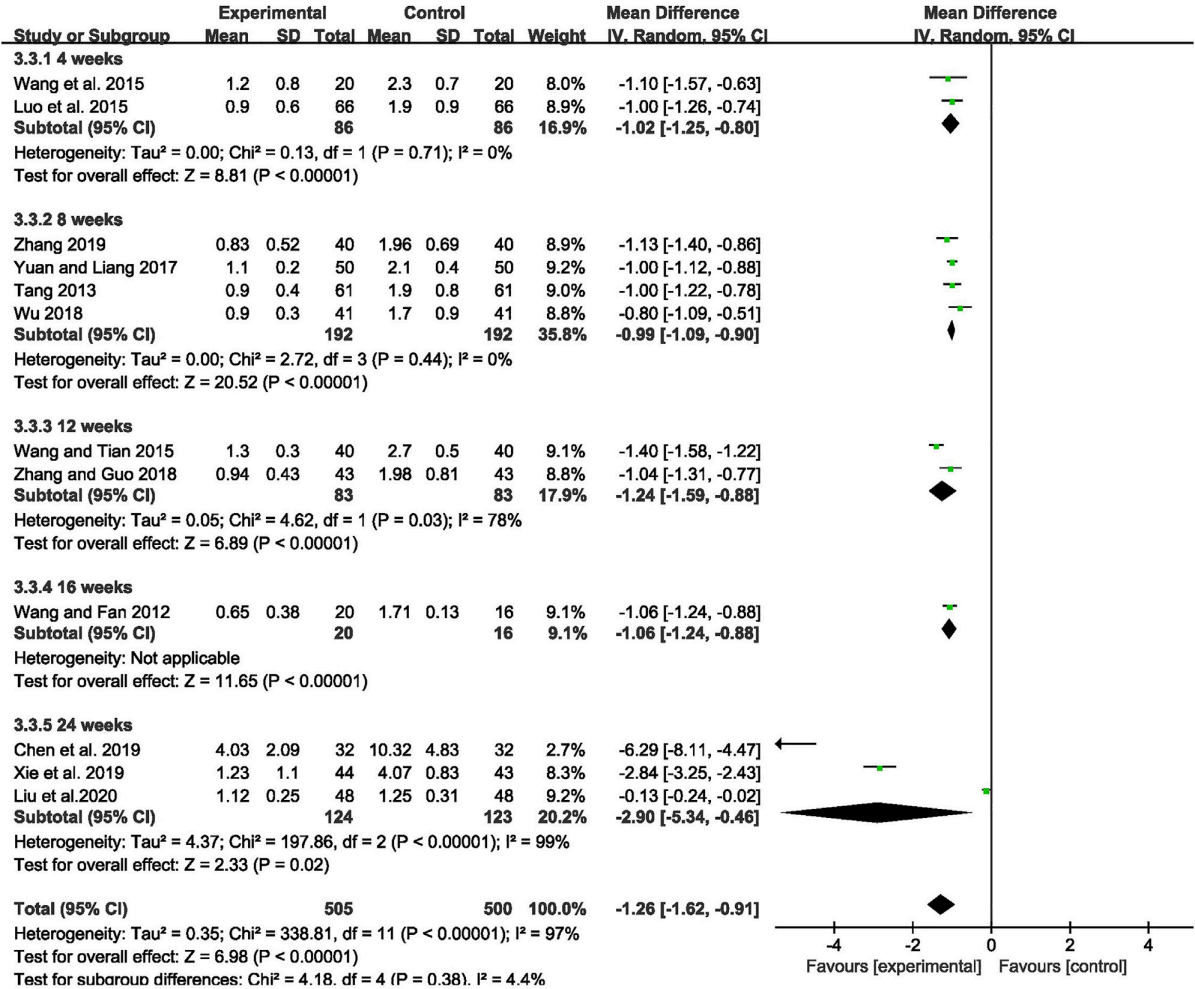
Forest plot displaying the results of subgroup analysis of CHM+AEDs vs. AEDs for monthly seizure frequency in patients with IE according to various treatment duration.

The outcome of monthly seizure frequency was reported in 12 RCTs (Wang and Fan, 2012; Tang, 2013; Luo et al., 2015; Wang Q et al., 2015; Wang and Tian, 2015; Yuan and Liang, 2017; Wu, 2018; Zhang and Guo, 2018; Chen et al., 2019; Xie et al., 2019; Zhang, 2019; Liu et al., 2020), including 1,005 patients with IE. Pooled results of these studies indicated that compared with AEDs alone, the combination of CHM and AEDs could remarkably reduce seizures by 1.26 per month in patients with IE [MD = −1.26, 95% CI (−1.62, −0.91), *p* < 0.00001, I^2^ = 97%] (as shown in Figure 3). In terms of the considerable heterogeneity, we further conducted a subgroup analysis to classify the treatment duration into 4 weeks [MD = −1.02, 95% CI (−1.25, −0.80), *p* < 0.00001], 8 weeks [MD = −0.99, 95% CI (−1.09, −0.90), *p* < 0.00001], 12 weeks [MD = −1.24, 95% CI (−1.59, −0.88), *p* < 0.00001], 16 weeks [MD = −1.06, 95% CI (−1.24, −0.88), *p* < 0.00001], and 24 weeks [MD = −2.90, 95% CI (−5.34, −0.46), *p* = 0.02]. Compared with AEDs alone, the combination of CHM and AEDs could remarkably reduce monthly seizure frequency, especially at 24 weeks (as shown in Figure 4). It indicated that a longer therapeutic course may lead to a more significant improvement.

### Abnormal Rate of EEG

**FIGURE 5:**
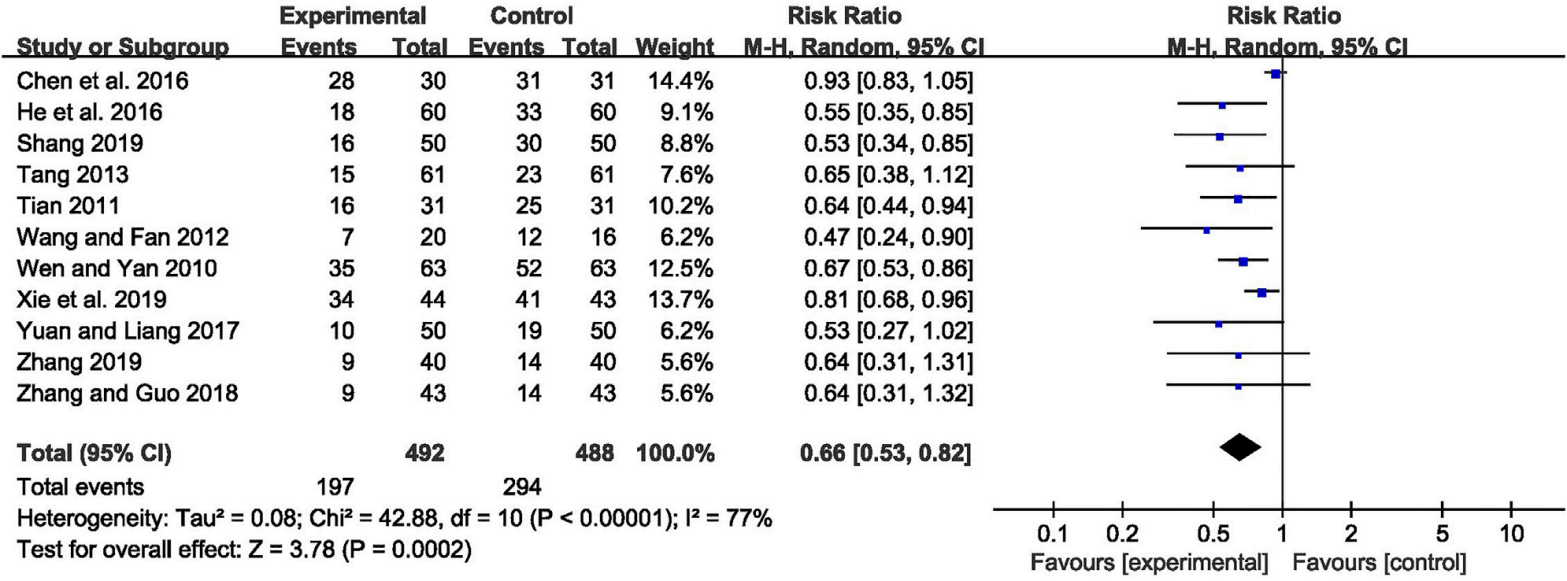
Forest plot displaying the effects of CHM+AEDs vs. AEDs for abnormal rate of EEG in patients with IE.

**FIGURE 6:**
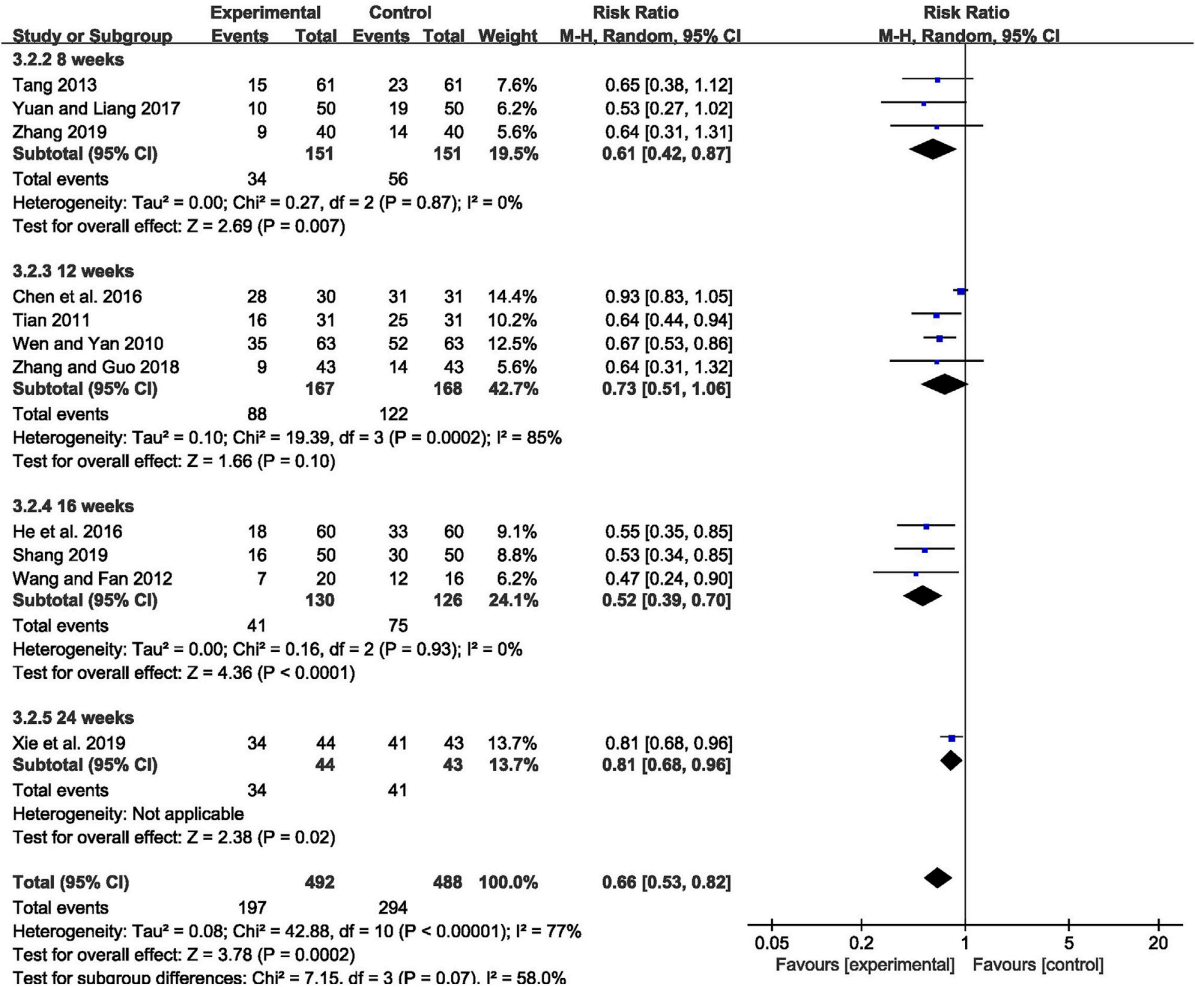
Forest plot displaying the results of subgroup analysis of CHM+AEDs vs. AEDs for abnormal rate of EEG in patients with IE according to various treatment duration.

The abnormal rate of EEG was recorded in 11 studies (Wen and Yan, 2010; Tian, 2011; Wang and Fan, 2012; Tang, 2013; Chen et al., 2016; He Q C et al., 2016; Yuan and Liang, 2017; Zhang and Guo, 2018; Shang, 2019; Xie et al., 2019; Zhang, 2019), including 980 patients. The meta-analysis showed that the abnormal rate of EEG was significantly lower in the CHM combined with AEDs group than in the AEDs alone group [RR = 0.66, 95% CI (0.53, 0.82), *p* = 0.0002; I^2^ = 77%] (as shown in Figure 5). According to the treatment duration, a subgroup analysis was conducted and the result showed that the abnormal rate of EEG was significantly lower in the CHM combined with AEDs group no matter the treatment duration lasted 8 weeks [RR = 0.61, 95% CI (0.42, 0.87), *p* = 0.007], or 16 weeks [RR = 0.52, 95% CI (0.39, 0.70), *p* < 0.0001], or 24 weeks [RR = 0.81, 95% CI (0.68, 0.96), *p* = 0.02] (as shown in Figure 6).

### Seizure Duration

**FIGURE 7:**
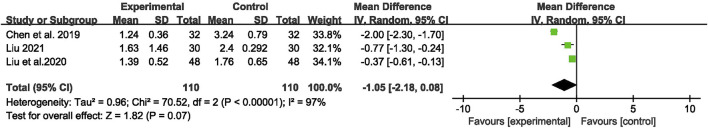
Forest plot displaying the effects of CHM+AEDs vs. AEDs for seizure duration in patients with IE.

Three studies (Chen et al., 2019; Liu et al., 2020; Liu, 2021) with 220 participants were included to evaluate the effect on seizure duration. Overall analyses revealed that there was no significant difference between the two groups [MD = −1.05, 95% CI (−2.18, 0.08), *p* = 0.07, I^2^ = 97%] (as shown in Figure 7).

### Quality of Life

**FIGURE 8:**
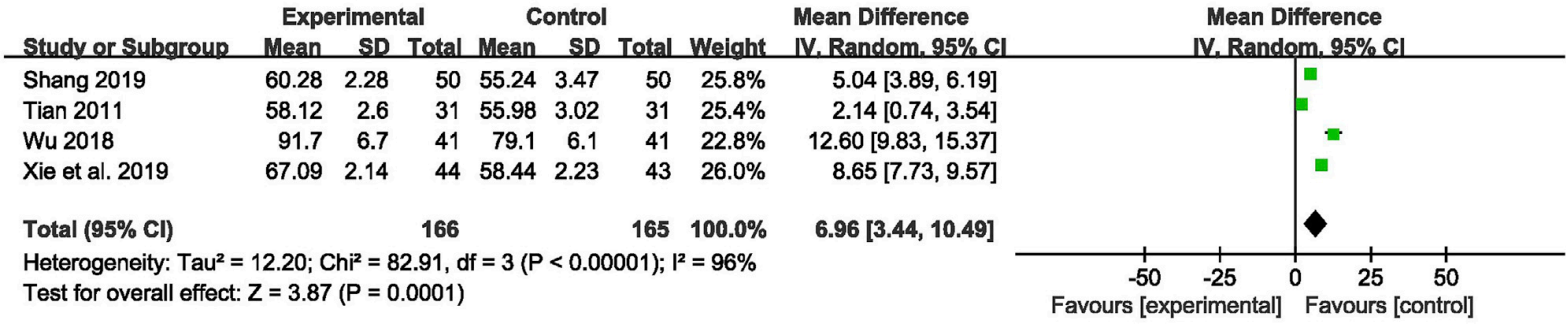
Forest plot displaying the effects of CHM+AEDs vs. AEDs for quality of life in patients with IE.

QoL was recorded in four studies (Tian, 2011; Wu, 2018; Shang, 2019; Xie et al., 2019) with 331 patients. The meta-analysis showed that participants taking CHM combined with AEDs had a significantly higher score on the QoL-31 scale than participants who were taking AEDs only [MD = 6.96, 95% CI (3.44, 10.49), *p* = 0.0001, I^2^ = 96%] (as shown in Figure 8).

### Adverse Events

**FIGURE 9:**
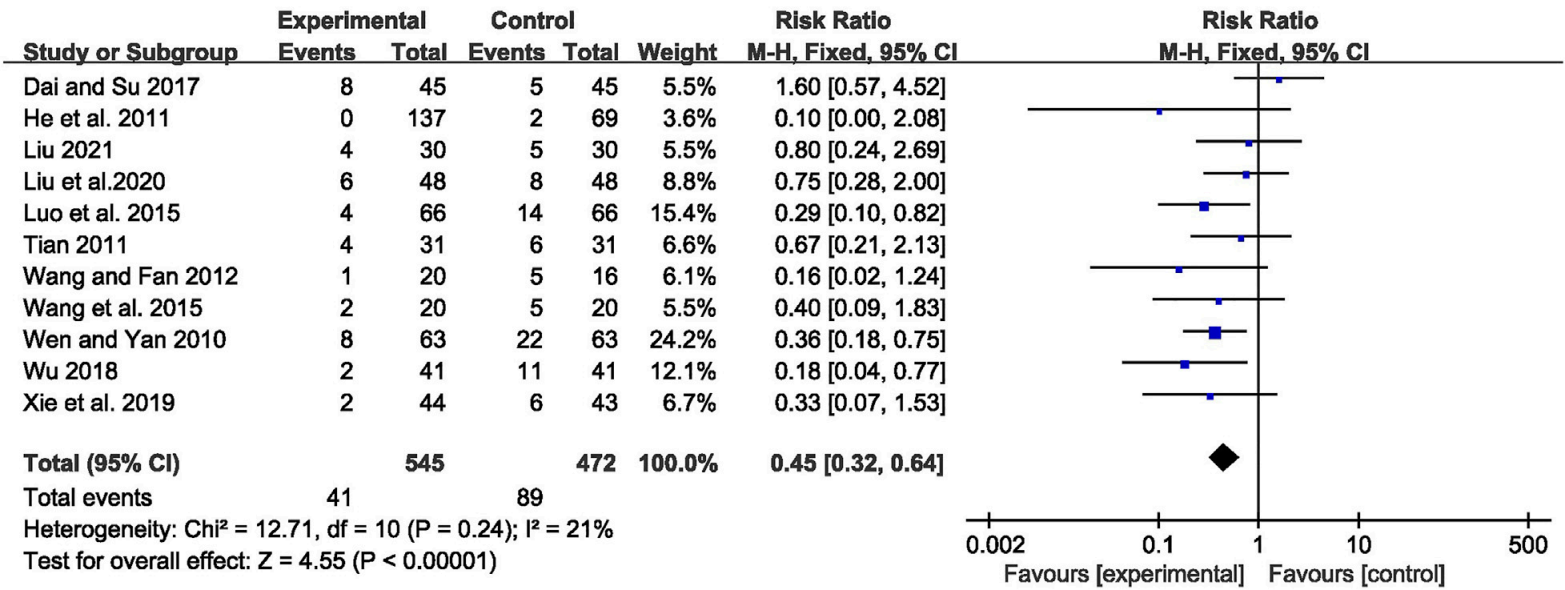
Forest plot displaying the effects of CHM+AEDs vs. AEDs for adverse events in patients with IE.

A total of 130 adverse events were reported in 11 trials (Wen and Yan, 2010; He et al., 2011; Tian, 2011; Wang and Fan, 2012; Luo et al., 2015; Wang Q et al., 2015; Dai and Su, 2017; Wu, 2018; Xie et al., 2019; Liu et al., 2020; Liu, 2021). Pooled results of these trials showed significantly fewer AEs in the CHM combined with AEDs group compared with the AEDs group, with no significant heterogeneity among studies [RR = 0.45, 95% CI (0.32, 0.64), *p* < 0.00001, I^2^ = 21%] (as shown in Figure 9).

### Meta-Regression

Considering the considerable heterogeneity among these 12 studies that reported monthly seizure frequency, a meta-regression analysis was performed to find the sources of heterogeneity. Numbers of subjects (regression coefficient β = 0.46, *p* = 0.664), treatment duration (regression coefficient β = −1.74, *p* = 0.132), interventions (regression coefficient β = 1.44, *p* = 0.199), JADAD scores (regression coefficient β = 0.14, *p* = 0.896) and diagnostic criteria (regression coefficient β = 0.16, *p* = 0.875) were taken as covariables, and the results indicated that the heterogeneity between studies had no significant correlation with the above covariables. As a result, the cause of the heterogeneity in this meta-analysis remains unknown, and the findings should be interpreted with caution (Supplementary Table S2).

### Sensitivity Analysis

A sensitivity analysis was performed by removing studies from the analysis one at a time to see how they affected the results. The findings revealed that no one study had a significant impact on the aggregated effect on monthly seizure frequency, abnormal EEG rate, or AEs, indicating that the findings were reasonably stable (Supplementary Figures S1–S3).

### Publication Bias

**FIGURE 10:**
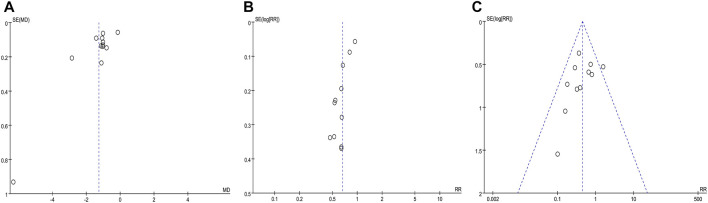
Funnel plot of the comparison between CHM combined with AEDs and AEDs alone for monthly seizure frequency **(A)**, abnormal rate of EEG **(B)**, and adverse events **(C)**.

Figures 10A–C were funnel plots which showed publication bias of the monthly seizure frequency, abnormal rate of EEG, and AEs in this meta-analysis, respectively. The funnel plots were visually asymmetrical, which indicated that there might be publication bias. Begge’s test and Egger’s test were also performed, and the results indicated possible publication bias in the abnormal rate of EEG (z = 0.31, *p* = 0.755; t = −5.81, *p* = 0.000) and monthly seizure frequency (z = 0.07, *p* = 0.945; t = −2.73, *p* = 0.021), and no publication bias for AEs (z = 1.4, *p* = 0.161; t = −1.18, *p* = 0.968). Despite the fact that the chance of publication bias in AEs was statistically tiny, we still believed there was a higher chance of it since the published literatures were all Chinese literature, and favorable outcomes were easier to publish.

### GRADE Evidence Profile

The GRADE method was used to explore the quality of evidence of the outcomes, which showed very low, low, and moderate quality with heterogeneity and methodological issues, indicating that these estimates were uncertain. Table 3 showed the GRADE evidence profile and a summary of the findings.

**TABLE 3:** GRADE summary of evidence for the effect of the combination of CHM and AEDs on intractable epilepsy.

Quality assessment							No of patients		Effect	Quality	Importance
No of studies	Design	Risk of bias	Inconsistency	Indirectness	Imprecision	Other considerations	CHM+AEDs	AEDs	MD/RR (95% CI)		
Monthly seizure frequency											
12	Randomized trials	Serious^1^	Serious^2^	Not serious	Not serious	Reporting bias^3^	505	500	MD −1.26 (−1.62, −1.26	⊕○○○ Very low	Critical
Abnormal rate of EEG											
11	Randomized trials	Serious^4^	Serious^2^	Not serious	Not serious	Reporting bias^3^	492	488	RR 0.66 (0.53, 0.82)	⊕○○○ Very low	Important
Seizure duration											
3	Randomized trials	Serious^4^	Serious^2^	Not serious	Serious^5^	None	110	110	MD −1.11 (−1.42, −1.11 (⊕○○○ Very low	Important
Quality of life											
4	Randomized trials	Serious^4^	Serious^2^	Not serious	Serious^3^	None	166	165	MD 6.96 (3.44, 10.49)	⊕○○○ Very low	Important
Adverse events											
11	Randomized trials	Serious^1^	Not serious	Not serious	Not serious	None	595	522	RR 0.45 (0.32, 0.64)	⊕⊕⊕○ Moderate	Important

AEDs, Antiepileptic drugs; CHM, Chinese herbal medicine; CI, Confidence interval; MD, mean difference; RR, Risk ratio.

1 All trials have mentioned applying a randomization methodology, but only some trials specified the method, only one of the trials recorded the methods of allocation concealment and the blindness.

2 Heterogeneity: I^2^ > 75%.

3 Publication bias.

4 All trials have mentioned applying a randomization methodology, but only some trials specified the method. No trials recorded the methods of allocation concealment and the blindness.

5 Small population size (<400).

## Discussion

IE is a potentially life-threatening disorder that affects about one-third of people who have epilepsy (Kwan and Brodie, 2000; Kwan et al., 2010). Modern medical studies have not clearly elucidated the mechanisms underlying IE, but its occurrence seems closely associated with multidrug transporters, pharmacokinetics, neural networks, and gene mutation (Tang et al., 2017). There is growing evidence that AEDs with a single mechanism of action show limitations in the treatment of IE. Therefore, AEDs with multiple mechanisms of action will be the new direction of IE treatment in the future. CHM has the advantages of multi-component and multi-target and offers a wealth of valuable experience in the management of epilepsy (Chen et al., 2016; Takayama et al., 2017; Wang et al., 2017). Meanwhile, researches in the last two decades have shown that CHM exhibited favorable pharmacological properties in the treatment of IE both *in vivo* and *in vitro* models (Kiasalari et al., 2013; Zhao et al., 2018; Yang et al., 2021). Due to the concerns about long-term therapy and adverse effects of AEDs, patients who cannot take AEDs might choose CHM, either used alone or in conjunction with AEDs. According to the 2015 Chinese Association of Anti-Epilepsy Guideline, the combination of AEDs with CHM in IE can achieve a synergistic effect. However, large numbers of scientific studies are still needed to provide sufficient evidence to support clinical application (China Association Against Epilepsy, 2015).

### Summary of Evidence

The most complete evaluation and meta-analysis of the effectiveness and safety of CHM coupled with AEDs for IE was undertaken in this systematic review and meta-analysis. In this systematic review, a total of 20 RCTs with 1,830 participants were included. It showed that the combination of CHM and AEDs could significantly reduce monthly seizure frequency and abnormal rate of EEG. However, there was no statistically significant difference in seizure duration between the two groups. As for safety, gastrointestinal discomfort, rash, dizziness, headache, drowsiness, and abnormal liver function were the most frequently reported AEs, which might be related to the side effects caused by AEDs. The combination of CHM and AEDs was safer compared to AEDs alone in terms of the incidence of AEs, which indicated that CHM might reduce the adverse reactions of AEDs. In addition, CHM combined with AEDs showed more advantages in improving the QoL. This outcome might be associated with the effect of CHM on improving the accompanying symptoms of IE patients and the adverse effects of AEDs, thus promoting physical function and overall health status. Through the subgroup analysis of monthly seizure frequency, we found that the efficacy might be positively correlated with the treatment duration, suggesting that a longer duration of treatment may achieve a better result. The results indicate that CHM is effective and safe as an adjunctive therapy for IE. In summary, CHM is valuable as a complementary and alternative therapy in the treatment of IE. However, due to the included RCTs are of low overall quality, the foregoing results must be confirmed in bigger samples of high-quality research.

Recent studies have revealed that high expression of multidrug resistance genes and their encoded proteins may be one of the mechanisms of drug resistance in patients with IE. The high expression of resistance proteins such as P-glycoprotein (P-gp) and breast cancer resistance protein (BCRP), the expression products of multi-drug resistance (MDR) genes, in the blood-brain barrier and brain tissue of patients with IE limits the concentration of AEDs in the lesion, resulting in a drug-resistant response (Seegers et al., 2002; Li et al., 2014). The mechanism of Chinese herbal medicine to control IE is still not fully understood. According to the multi-target effect of Chinese herbal medicine, it may increase the concentration of AEDs in the epileptic focus by inhibiting the high expression of various drug-resistant proteins in the brain (Xie, 2011; Jia et al., 2018), or it may improve the efficacy of antiepileptic drugs by regulating the balance of excitatory and inhibitory neurotransmitters in the lesioned brain (Yan, 2014). Several studies have found that Chinese herbal medicine can inhibit the expression of MDR1 mRNA and multidrug resistance associated protein 1 (MRP1) mRNA in the hippocampal CA1, dentate gyrus, and temporal lobe, and inhibit the drug transport function of P-gp and MRP1, thus reversing the drug resistance of AEDs, thus playing a synergistic effect (Wang-Tilz et al., 2006; Chen et al., 2013; Yan et al., 2015; Li et al., 2016; Liang et al., 2016). Furthermore, Chinese herbal medicine can inhibit the effect of P-glycoprotein in rat brain microvascular endothelial cells and regulate the permeability of the blood-brain barrier by downregulating the high expression of pregnane X receptor induced by recurrent seizures and application of AEDs, thus increasing the concentration of AEDs in brain tissues and inhibiting epilepsy drug resistance (Wang, 2018). In addition, studies have shown that Chinese herbal medicine can increase the efficacy of synergistic treatment of IE by down-regulating the expression of BCRP and BCRP mRNA in rat brain microvascular endothelial cells and decreasing the value of glutamic acid/γ-aminobutyric acid in the extracellular fluid of the hippocampal CA3 area, which can sensitize antiepileptic drugs (Brodie and Sills., 2011; Sirven et al., 2012; Wang Q et al., 2015).

On the other hand, neuroinflammation is now considered to be a potential pathogenesis of IE as well (Vezzani and Viviani., 2015). Some studies have found that Chinese herbal medicine has certain immunosuppressive effects, which can reduce neuroinflammation by inhibiting microglia activation and decreasing the level of pro-inflammatory cytokines, thereby reducing neuronal death and suppressing seizures (Zhang et al., 2020). In addition, Chinese medicine can also block sodium channels by reducing sodium channel currents, enhancing sodium channel inactivation, and inhibiting recovery after inactivation, thereby reducing recurrent spontaneous seizures in epilepsy, which may be one of the mechanisms of its action in the treatment of IE rats (Wang et al., 2014; Yan et al., 2014; Fang et al., 2018; Liu et al., 2019; Nie et al., 2020; Liu et al., 2021). Whether the efficacy of Chinese herbal medicine combined with antiepileptic drugs for intractable epilepsy can be explained by these mechanisms remains to be fully explored.

### Comparison With Previous Studies

Only one systematic review and meta-analysis published in China has demonstrated the efficacy and safety of combining CHM with AEDs for IE before, which involved 16 RCTs with 1,444 patients, revealed that combining CHM with AEDs was effective in increasing the total effective rate and reducing the abnormal rate of EEG in the treatment of IE (Zhou and Mao, 2019). This meta-analysis has some advantages in comparison with the previous meta-analysis. Firstly, for the outcome measures, total effective rate was adopted as the primary outcome in previous meta-analysis, which had varied definitions and therefore should not be pooled and might cause undetectable heterogeneity in the meta-analysis. Therefore, this meta-analysis abandoned the total effective rate and used the monthly seizure frequency as the primary outcome, which is a more internationally recognized indicator for the evaluation of epilepsy. Notably, two new clinical outcomes (QoL and seizure duration) were added to the analysis to make the results more comprehensive. Secondly, the previous systematic review included some studies that we excluded due to similar data and incomplete reports, which tend to be rated with a high risk of bias. We used more stringent and clearer criteria to reduce the degree of heterogeneity. Thirdly, we searched more databases, and the search strategy was more comprehensive. Twenty RCTs were recruited in this meta-analysis, including several newly-published (especially those published between 2019 and 2021) and well-conducted trials. Fourthly, in the meta-analysis, we performed the subgroup analysis to make the results more stable.

This meta-analysis showed that the combination of CHM and AEDs could significantly reduce abnormal rate of EEG and decrease the adverse events in patients with IE, which were similar to the previous study (Zhou and Mao, 2019). In addition, there were new findings in the present meta-analysis that the combination of CHM and AEDs could significantly reduce the monthly seizure frequency and improve quality of life of patients, which were not involved in the previous meta-analysis. This meta-analysis underwent a more comprehensive, up-to-date, and PRISMA-compliant systematic review, and the results of the meta-analysis were more reliable.

### Strengths and Limitations

This meta-analysis provided a comprehensive review of all studies on this issue and summarized the evidence supporting the efficacy and safety of CHM in combination with AEDs for the treatment of IE. Considering the limitations of AEDs in the treatment of IE and that many patients already use CHM as adjuvant therapy, this topic has great clinical relevance. The strengths of this systematic review and meta-analysis included a clear research question and a precise study protocol, which helped reduce selection bias. All of the included studies were RCTs, which helped to avoid the limitations of non-randomized studies such as recall or selection bias. Additionally, the number of trials and the overall sample size were comparatively large (20 trials with 1,830 patients). Subgroup assessments and meta-regression evaluations were performed to identify the source of heterogeneity. Sensitivity estimation revealed that the outcomes of this meta-analysis were found to be relatively robust.

This systematic review was subject to some limitations. We found that in most RCTs, only the administration of AEDs to patients was described, but the name of the drug and the method of administration were not mentioned in detail. Although only RCTs were included, these studies still had some inherent methodological flaws: 1) All of the RCTs in this meta-analysis were done in China, which may restrict the generalizability of these findings. 2) Only 11 studies described the method of random sequence generation, and only 1 trial was double-blind and placebo-controlled. The poor methodological quality could lead to an inflated effect on efficacy and a lower dependability of the evidence, consequently diminishing the credibility of the results. 3) Only 4 of all the included studies described subject loss to follow-up or dropout, and only 1 of these studies performed an intention-to-treat analysis for this issue. 4) Selection bias and publication bias may exist because all studies did not register and publish study protocols in advance. 5) Considering the small number of included studies and the limited sample size of some studies, the results should be regarded with caution. 6) Despite the fact that sensitivity analysis verified the reliability of these findings, there was substantial heterogeneity in several of them. As a result, these heterogeneous results should be viewed with care. Furthermore, most of the included studies did not describe the type of seizures, so it was not possible to perform subgroup analyses by different type of seizures, and therefore possible differences in outcomes arising from the subgroup factor could not be assessed. Finally, since IE is a chronic disease that may fluctuate over a long course, ongoing follow-up is important to ascertain the real effectiveness and long-term effects of the combination of CHM and AEDs.

### Implication for Research

The quality of RCTs included in the meta-analysis had an important impact on the credibility of the meta-analysis results. Therefore, more high-quality RCTs are needed to assess the efficacy and safety of CHM combined with AEDs for the treatment of IE. Some recommendations for further research are made based on the aforementioned limitations: 1) Registration of the study protocol should be done on the clinical trials registry platform in advance. 2) The sample size estimation method should be described in detail. 3) The methodological quality, including randomization, allocation concealment, and blinding, should be described in detail. Placebo controls should be used whenever possible. 4) Complete reporting of outcome data and analysis of reasons for dislodgement and withdrawal. 5) A large sample size and long-term follow-up are required to evaluate the efficacy and safety of CHM combined with AEDs in IE and provide evidence-based data for promoting the use of CHM. 6) To ensure the scientific quality and rigor of studies, RCTs should be designed and clinical trial results should be reported in strict conformity with the CONSORT statement’s standard (Bian et al., 2011). Furthermore, exploring the effect of the combination of CHM and AEDs for different type of seizures, and whether there are differences in monthly seizure frequency between different type of seizures are other problems we will face in the future.

## Conclusion

The combination of CHM and AEDs could improve seizure control by reducing monthly seizure frequency and abnormal rate of EEG with a decreased risk of adverse events in patients with IE. However, these findings must be interpreted carefully due to the high or uncertain risk of bias in the included trials. To provide stronger evidence for the use of CHM combined with AEDs in IE, high-quality RCTs will be urgently warranted in the future.

## Abbreviations

AEs, adverse events; AEDs, antiepileptic drugs; CHM, Chinese herbal medicine; CI, confidence interval; CONSORT, Consolidated Standards for Reporting Trials; EEG, electroencephalogram; GRADE, grading of recommendations assessment, development, and evaluation; IE, intractable epilepsy; MD, mean difference; PRISMA, Preferred Reporting Items for Systematic reviews and Meta-Analyses; QoL, quality of life; QOLIE-31, Quality of Life in Epilepsy -31; RCTs, randomized controlled trials; RR, risk ratio.
