# Supplementary material for: Chinese Herbal Medicine Combined With Antiepileptic Drugs for Intractable Epilepsy: A Systematic Review and Meta-Analysis of Randomized Controlled Trials
Source: Front Pharmacol. 2022 Jul 20;13:917099. doi: 10.3389/fphar.2022.917099 (PMC10117129; doi:10.3389/fphar.2022.917099)

Supplementary Online Content

Ying Zhao, Jialin Liu, Qingxuan Liu, Mengwei Wu, Siyuan Yuan, Weiwei Xu, Ying Wang, Kaiyue Wang, Lili Li, Hufang Zhou, Jinmin Liu. Chinese Herbal Medicine Combined With Antiepileptic Drugs for Intractable Epilepsy: A Systematic Review and Meta-Analysis of Randomized Controlled Trials

eMethod. Search Strategy

eTable 1. List of studies excluded at full-text screening stage

eTable 2. Meta-regression of sources of heterogeneity in included studies which reported monthly seizure frequency

eFigure 1. Sensitivity analysis of monthly seizure frequency in patients with IE treated by combination of CHM and AEDs

eFigure 2. Sensitivity analysis of abnormal rate of EEG in patients with IE treated by combination of CHM and AEDs

eFigure 3. Sensitivity analysis of adverse events in patients with IE treated by combination of CHM and AEDs

e Methods. Search Strategy

Search strategy for Medline Database (September 14, 2021)

#1 "Medicine, Chinese Traditional"[Mesh]

#2 "Drugs, Chinese Herbal"[Mesh]

#3 ((((((((((((((traditional Chinese medicine[Title/Abstract]) OR (TCM[Title/Abstract])) OR (Chinese herbal compound[Title/Abstract])) OR (Herbal medicine[Title/Abstract])) OR (Chinese patent medicine[Title/Abstract]))) OR (Chinese herbal[Title/Abstract])) OR (Chinese medicine[Title/Abstract])) OR (Chinese proprietary medicine[Title/Abstract])) OR (Chinese proprietary drug[Title/Abstract])) OR (Chinese patent drug[Title/Abstract])) OR (complementary[Title/Abstract])) OR (alternative medicine[Title/Abstract])) OR (CAM[Title/Abstract])

#4 #1 OR #2 OR #3

#5 "Drug Resistant Epilepsy"[Mesh]

#6 ((((((((((((Drug Resistant Epilepsy[Title/Abstract]) OR (Drug Resistant Epilepsies[Title/Abstract])) OR (Medication Resistant Epilepsy[Title/Abstract])) OR (Medication Resistant Epilepsies[Title/Abstract])) OR (Intractable Epilepsy[Title/Abstract])) OR (Intractable Epilepsies[Title/Abstract])) OR (Refractory Epilepsy[Title/Abstract])) OR (Refractory Epilepsies[Title/Abstract])) OR (Drug Refractory Epilepsy[Title/Abstract])) OR (Drug Refractory Epilepsies[Title/Abstract])) OR (Medically refractory epilepsy[Title/Abstract])) OR (drug-resistant epilepsy[Title/Abstract])) OR (temporal lobe epilepsy[Title/Abstract])

#7 #5 OR #6

#8 randomized controlled trial [pt]

#9 controlled clinical trial [pt]

#10 randomized [tiab]

#11 placebo [tiab]

#12 drug therapy [sh]

#13 randomly [tiab]

#14 trial [tiab]

#15 groups [tiab]

#16 #8 OR #9 OR #10 OR #11 OR #12 OR #13 OR #14 OR #15

#17 animals [mh]) NOT humans [mh])

#18 #16 NOT #17

#19 #4 AND #7 AND #18

e Table 1. List of studies excluded at full-text screening stage

Number	Author	Title	Reason
1	Bo, L. K.	Therapeutic effect observation on the treatment of 64 cases of intractable epilepsia major with adjuctive therapy of Shenpu Decoction	No outcome data for extraction
2	Shu, J.	Clinical observation on adjuvant treatment of refractory epilepsy with Chaibei Zhixian Decoction	No outcome data for extraction
3	Nie, L. Y.	Clinical study of the combination of Chaibei Zhixian Decoction with anti-epilepsy drugs in the treatment of intractable epilepsy complex partial seizures	No outcome data for extraction
4	Yang, M. Q.	To analyze the safety of Chaibei Zhixian Decoction in treating refractory epilepsy	No outcome data for extraction
5	Wang, B.	Clinical observation of Xuefu Zhuyu Decoction in treating intractable epilepsy caused by brain trauma	No outcome data for extraction
6	Zhang, J.	Clinical observation on refractory epilepsy treated by integrated Traditional Chinese and Western medicine	No outcome data for extraction
7	He, X. Y.	Clinical observation on treating intractable epilepsy with integrated traditional Chinese and Western Medicine	No outcome data for extraction
8	Wu, Y.	Clinical observation on refractory epilepsy treated by integrated Traditional Chinese and Western medicine	No outcome data for extraction
9	Xiao, J. F.	Clinical observation on refractory epilepsy with heart-kidney-yang deficiency treated by integrated Chinese and Western medicine	No outcome data for extraction
10	Chen, C. P.	Clinical observation of Chaihu Shugan Decoction combined with Guipi Decoction in treating refractory epilepsy syndrome of Liver depression and spleen deficiency	No outcome data for extraction
11	Wang, X. H.	Randomized controlled trial of Chaibei Zhixian Granules in the treatment of refractory epilepsy	No outcome data for extraction
12	Wang, Q. X.	Clinical observation on 78 cases of refractory epilepsy treated with Zhenhuang Pingxian Pill and Dianjianan	No outcome data for extraction
13	He, Q. C.	Effect of Chaihu Shugan decotion for intractable epilepsy and curative effect of related factors	No outcome data for extraction

14	Liu, Y. X.	Clinical analysis of 72 epileptic patients treated with alkaline extract of Euphorbia fisheriana	Participants did not meet the inclusion criteria
15	Sun, Y. D.	Clinical observation of Shaoxing Zhixian tablet in the treatment of epilepsy	Participants did not meet the inclusion criteria
16	Xiao, H. Q.	Effect of Bupleurum plus Longgu Oyster Decoction on SOD and MDA in patients with temporal lobe epilepsy	Participants did not meet the inclusion criteria
17	Yang, X. H.	Efficacy and safety analysis of Bupleurum plus Longgu Oyster Decoction combined with oxazepine in the treatment of temporal lobe epilepsy	Participants did not meet the inclusion criteria
18	Zhou, B.	Effects of Bupleurum plus Longgu Oyster Decoction on EEG and serum NGF and BDNF levels in patients with temporal lobe epilepsy	Participants did not meet the inclusion criteria
19	Li, H. X.	Clinical observation of Jiawei Chaihu Shugan Decoction combined with carbamazepine in the treatment of temporal lobe epilepsy with qi stagnation and spittoon coagulation	Participants did not meet the inclusion criteria
20	Chen, A. L.	Clinical observation of modified Chaihu Shugan Powder combined with carbamazepine in the treatment of eclampsia with wind-phlegm closure	Participants did not meet the inclusion criteria
21	Cao, J.	Study on the quality of life and seizure control of temporal lobe epilepsy treated by resolving phlegm and dispelling wind	Participants did not meet the inclusion criteria
22	Zhang, J. D.	Clinical observation of 102 cases of refractory epilepsy treated by integrated traditional Chinese and western medicine	Participants did not meet the inclusion criteria
23	Wang, B. J.	Clinical analysis of Chinese and western combined comprehensive treatment for refractory epilepsy	Interventions did not meet the inclusion
24	Fan, W. T.	Clinical observation on the treatment of refractory epilepsy with Chinese and western integrated treatment	Interventions did not meet the inclusion
25	Wu, H. M.	Antioxidant and Anticonvulsant Effect of a Modified Formula of Chaihu-Longu-Muli-Tang	not RCT
26	Xiao, D. P.	Clinical observation of Pinggan Zhixian Prescription combined with western medicine in the treatment of refractory epilepsy	not RCT

27	Wu, C. H.	A randomized parallel-controlled study of Chaibei Zhixian Decoction combined with western medicine in the treatment of complex partial seizures of refractory epilepsy	not RCT
28	Gao, Y.	Effect analysis of Xuefu Zhuyu Decoction on refractory epilepsy caused by brain trauma	not RCT
29	Deng, Y.	30 cases of Zhixian Decoction in the treatment of refractory epilepsy	not RCT
30	Huang, Y. J.	Clinical observation on 118 cases of refractory epilepsy treated by integrated traditional Chinese and western medicine	not RCT
31	Huang, Y. S.	Observation on curative effect of Chaihu Shugan Decoction in treatment of 108 cases of primary epilepsy	not RCT
32	Liu, Z. F.	Clinical observation on the treatment of refractory grand epilepsy by Xianfukang	not RCT
33	Xue, X. H.	Clinical observation of 85 cases of refractory epilepsy treated by Chinese medicine "Diankexing"	not RCT
34	Zuo, T. X.	Clinical and experimental study of "Zhixian Decoction" in treating refractory epilepsy	not RCT
35	Chen, M.	Clinical observation on treating 61 cases of refractory epilepsy with Qutan Dingxian Decoction	not RCT
36	Okabe, T.	Clinical observation of the use of herbal medicine together with anti-convulsive medicine for intractable epilepsy	not RCT
37	Wang, X. H.	Randomized controlled trial of adding Chaibei Zhixian granules in the treatment of refractory epilepsy and its mechanism on P-GP and PXR	duplicate publication
38	Zhang, Y. G.	Observation on curative effect of refractory epilepsy treated by integrated traditional Chinese and western medicine	duplicate publication
39	Chen, C. P.	Clinical observation of Chaihu Shugan Decoction combined with Guipi Decoction in treating refractory epilepsy syndrome of Liver depression and spleen deficiency	duplicate publication

40	Yu, J. B.	Clinical study on treating refractory epilepsy with Qingxin Wendan Decoction	duplicate publication
41	He, Q. C.	Effect of Chaihu Shugan Decoction on refractory epilepsy and related factors of curative effect	duplicate publication
42	Wang, H. X,	Clinical analysis of combined Chinese and western treatment of refractory epilepsy	full text unavailable
43	Yin, L. P.	100 cases of intractable epilepsy treated by integrated traditional Chinese and western medicine	full text unavailable
44	Liu, Z. F.	Clinical and experimental study of Zhixian Decoction in treating refractory epilepsy (grand seizure)	full text unavailable
45	Wang, W. M.	Clinical study on repairing abnormal perfusion foci of epileptic patients with traditional Chinese medicine assisted antiepileptic drugs	full text unavailable
46	Wu, Y.	Clinical observation on refractory epilepsy treated by integrated Traditional Chinese and Western medicine	Used mistaken data
47	He, X. Y.	Clinical observation on treating intractable epilepsy with integrated traditional Chinese and Western Medicine	Used mistaken data

Abbreviations: IE, intractable epilepsy; RCTs, randomized controlled trials.

e Table 2. Meta-regression of sources of heterogeneity in included studies which reported monthly seizure frequency

	β	95% CI		*P* vlue
		Lower Limit	Upper Limit	
numbers of subject	0.46	-0.03	0.05	0.664
treatment duration	-1.74	-1.01	0.17	0.132
interventions	1.44	-0.41	1.57	0.199
JADAD scores	0.14	-1.64	1.84	0.896
diagnostic criteria	0.16	-0.55	0.63	0.875

Abbreviations: CI, confidence intervals.

e Figure 1. Sensitivity analysis of monthly seizure frequency in patients with IE treated by combination of CHM and AEDs

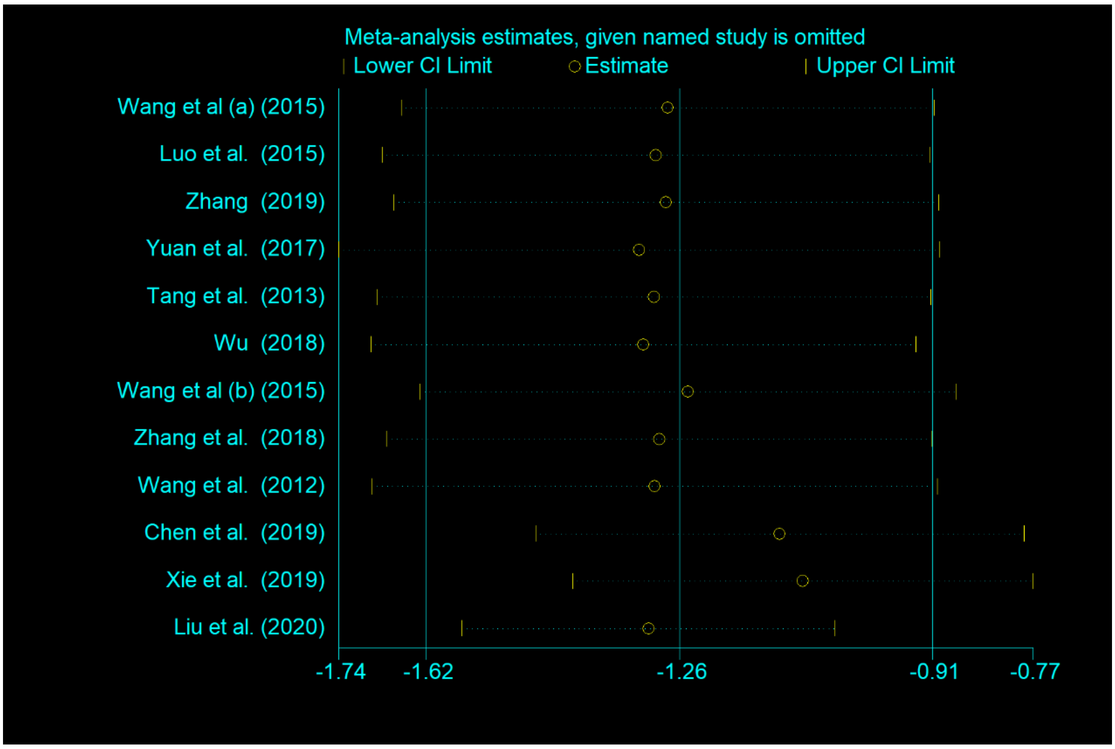

e Figure 2. Sensitivity analysis of abnormal rate of EEG in patients with IE treated by combination of CHM and AEDs

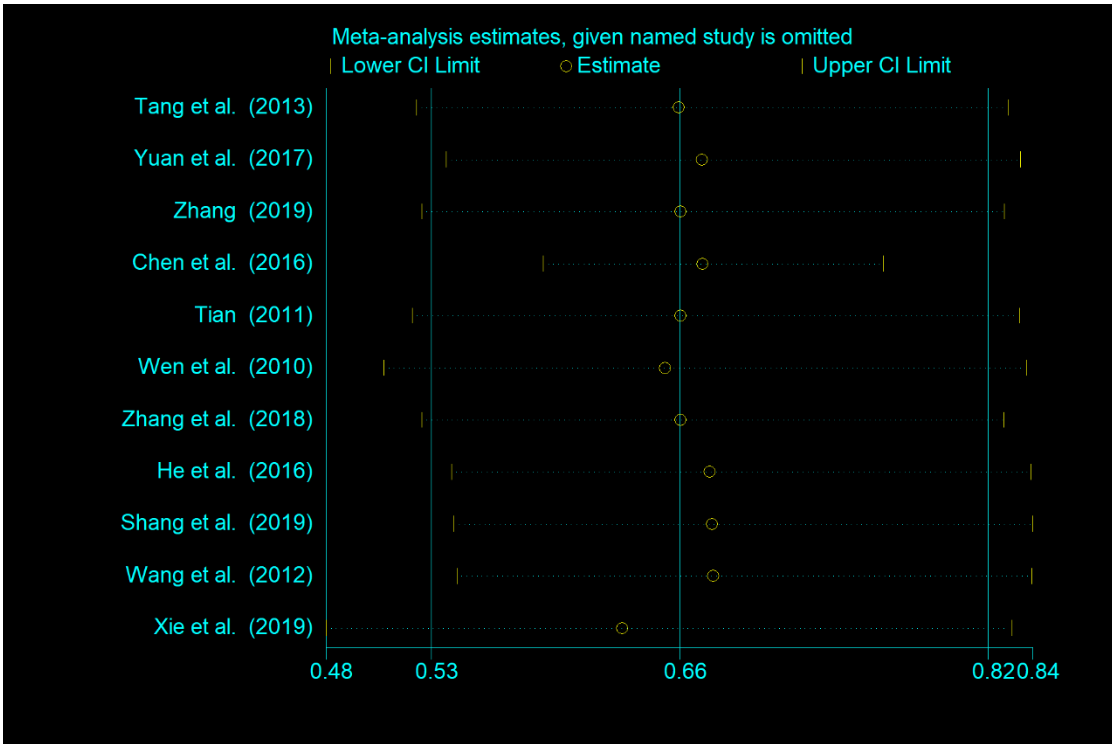

e Figure 3. Sensitivity analysis of adverse events in patients with IE treated by combination of CHM and AEDs

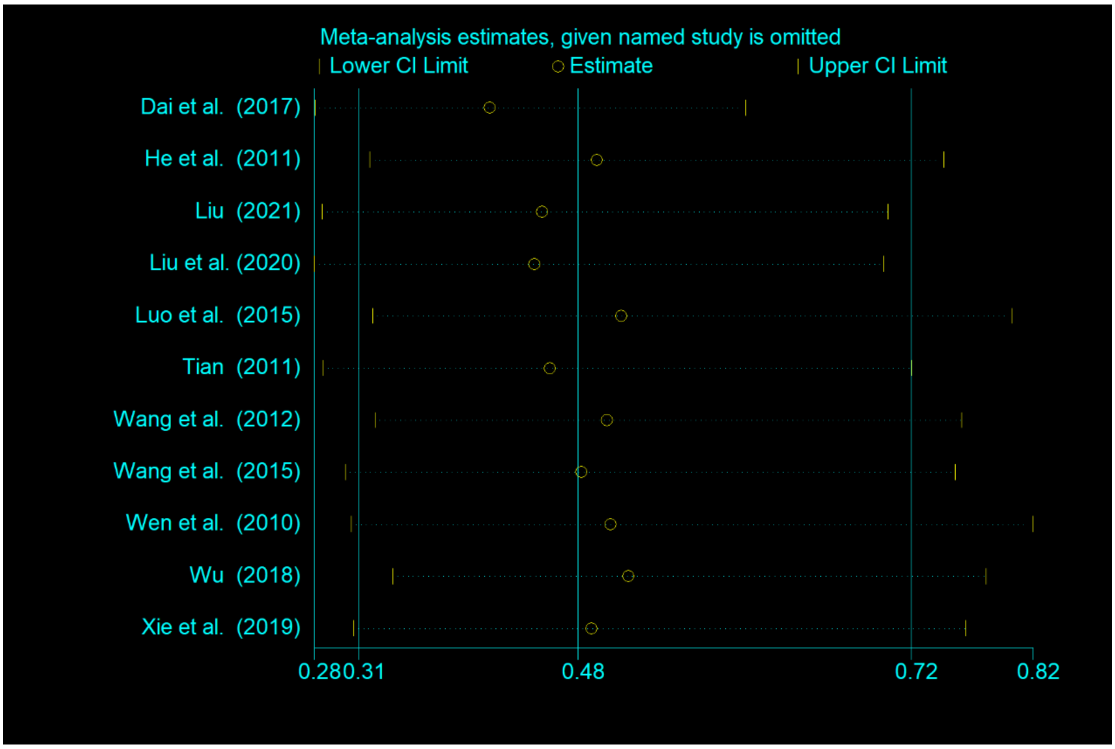

Supplement: Supplementary file 1 [file DataSheet1.PDF]
